# Spatial-Selective Volumetric 4D Printing and Single-Photon Grafting of Biomolecules within Centimeter-Scale Hydrogels via Tomographic Manufacturing

**DOI:** 10.1002/admt.202300026

**Published:** 2023-05-23

**Authors:** Marc Falandt, Paulina Nuñez Bernal, Oksana Dudaryeva, Sammy Florczak, Gabriel Gröfibacher, Matthias Schweiger, Alessia Longoni, Coralie Greant, Marisa Assunção, Olaf Nijssen, Sandra van Vlierberghe, Jos Malda, Tina Vermonden, Riccardo Levato

**Affiliations:** Department of Clinical Sciences Faculty of Veterinary Medicine Utrecht University Utrecht 3584CT, The Netherlands; Department of Orthopedics University Medical Center Utrecht Utrecht University Utrecht 3584CX, The Netherlands; Department of Clinical Sciences Faculty of Veterinary Medicine Utrecht University Utrecht 3584CT, The Netherlands; Department of Orthopedics University Medical Center Utrecht Utrecht University Utrecht 3584CX, The Netherlands; Polymer Chemistry & Biomaterials Group Centre of Macromolecular Chemistry Department of Organic & Macromolecular Chemistry Faculty of Sciences Ghent University Ghent 9000, Belgium; BIO INX BV Technologiepark-Zwijnaarde 66, Ghent 9052, Belgium; Department of Orthopedics University Medical Center Utrecht Utrecht University Utrecht 3584CX, The Netherlands; Department of Clinical Sciences Faculty of Veterinary Medicine Utrecht University Utrecht 3584CT, The Netherlands; Polymer Chemistry & Biomaterials Group Centre of Macromolecular Chemistry Department of Organic & Macromolecular Chemistry Faculty of Sciences Ghent University Ghent 9000, Belgium; BIO INX BV Technologiepark-Zwijnaarde 66, Ghent 9052, Belgium; Department of Clinical Sciences Faculty of Veterinary Medicine Utrecht University Utrecht 3584CT, The Netherlands; Department of Orthopedics University Medical Center Utrecht Utrecht University Utrecht 3584CX, The Netherlands; Department of Pharmaceutical Sciences Faculty of Science Utrecht University Utrecht 3584CG, The Netherlands; Department of Clinical Sciences Faculty of Veterinary Medicine Utrecht University Utrecht 3584CT, The Netherlands; Department of Orthopedics University Medical Center Utrecht Utrecht University Utrecht 3584CX, The Netherlands

**Keywords:** 4D printing, biofabrication, light-based printing, photopatterning, volumetric additive manufacturing

## Abstract

Conventional additive manufacturing and biofabrication techniques are unable to edit the chemicophysical properties of the printed object postprinting. Herein, a new approach is presented, leveraging light-based volumetric printing as a tool to spatially pattern any biomolecule of interest in custom-designed geometries even across large, centimeter-scale hydrogels. As biomaterial platform, a gelatin norbornene resin is developed with tunable mechanical properties suitable for tissue engineering applications. The resin can be volumetrically printed within seconds at high resolution (23.68 ± 10.75 μm). Thiol–ene click chemistry allows on-demand photografting of thiolated compounds postprinting, from small to large (bio)molecules (e.g., fluorescent dyes or growth factors). These molecules are covalently attached into printed structures using volumetric light projections, forming 3D geometries with high spatiotemporal control and ≈50 μm resolution. As a proof of concept, vascular endothelial growth factor is locally photografted into a bioprinted construct and demonstrated region-dependent enhanced adhesion and network formation of endothelial cells. This technology paves the way toward the precise spatiotemporal biofunctionalization and modification of the chemical composition of (bio)printed constructs to better guide cell behavior, build bioactive cue gradients. Moreover, it opens future possibilities for 4D printing to mimic the dynamic changes in morphogen presentation natively experienced in biological tissues.

## 1 Introduction

3D printing technologies have rapidly become fundamental tools for biomedical research and personalized implant generation. These technologies have exceptional ability to generate biomaterial-based constructs with customized architecture and precise spatial patterning of different biocompatible materials and living cells (i.e., via biofabrication technologies including bioprinting).,^[1,2]^ Key applications of biofabricated structures that mimic salient features of native tissues include patient-specific in vitro models for drug discovery, and implantable constructs for regenerative medicine.^[3]^

A main limitation of current bioprinting technologies is the lack of using stimuli-responsive materials and shape memory polymers as building blocks. These approaches have often been defined as 4D printing, with time being the fourth dimension.^[4]^ Typically, these strategies include the induction of predictable and desired changes in stiffness, architecture, or size of constructs postprinting upon exposure to heat,^[5]^ ions,^[6]^ ultrasounds,^[7]^ or electromagnetic fields.^[8]^ These geometrical changes are especially useful in the field of soft robotics,^[9]^ and to mechanically stimulate cells during tissue culture.^[10]^ On the other hand, time-dependent, on-demand modifications of the biochemical properties of the printed structure remain particularly challenging. Precise spatial control over the biochemical composition of a construct would allow for the gradual presentation of different growth factors and morphogens into local cell environments, thus enabling local control of (stem) cell fate, mimicking environmental changes naturally occurring during developmental, healing, and degenerative processes. In the field of 3D printed hydrogel for tissue engineering applications, capturing the physicochemical composition of the native extracellular matrix (ECM) remains an important objective. In fact, tissue ECM displays unique region-dependent mechanical properties, and it also acts as a depot of biologically active biochemical signals. These are both in the form of peptide sequences embedded in the ECM polymeric backbone, as well as through the release and presentation of growth factors tethered to specific domains in ECM proteins and glycans. Several elegant hydrogel-based systems have been designed to be readily functionalized with such bioactive sequences during their preparation. Often, these systems result in the isotropic distribution of bioactive cues that are effective in steering cell behavior.^[11,12]^ Alternatively, postcuring in photopolymers can be leveraged to graft molecules of interest onto prefabricated structures, homogenously in specific regions in which the still reactive material is present, as shown, for example, in studies processing nonhydrogel materials.^[13,14]^

To date, spatioselective chemical grafting of bioactive molecules has been typically performed in tissue cultures exploiting the contactless nature of light-based fabrication technologies, for example, with lithographic techniques,^[15–17]^ which permit projections of 2D patterns, and via multiphoton lithography.^[18,19]^ The latter, albeit showing exceptionally high resolution (<1 μm), is limited by the working distance of the objective used in the device, which rarely exceeds 1 mm, thus preventing photochemical editing of larger objects.

In the present study, we introduce a new visible-light-mediated technology to precisely imprint volumetric 3D patterns of fluorescent moieties and biological molecules within cubic-centimeter-scale hydrogels, leveraging the potential of tomographic printing. This approach enables the generation of geometrically defined patterns of biologically active species for directing cell behavior, which can be introduced arbitrarily at any time point after hydrogel cross-linking and printing.

Previously, our group demonstrated the possibility to produce complex, hydrogel-based cell-free or cell-laden constructs of clinically relevant size in mere seconds, via volumetric bioprinting (VBP).^[20]^ This novel light-based printing method, inspired by computed tomography, generates whole objects in a layerless fashion (as opposed to conventional layer-by-layer 3D printing).^[20–22]^ This permits high-speed printing, while still achieving printing resolution in the range of ≈40–50 μm, even when printing in the presence of cells and complex cellular aggregates like organoids.^[23]^ In volumetric printing (VP), also called volumetric additive manufacturing, a digital micromirror device shapes (visible) light into filtered backprojections of the object to be printed, as instructed by a tomographic reconstruction algorithm. The projections are sent to a rotating volume of a photoresponsive material at specific angles, and the resulting light dose accumulation allows for selective cross-linking of the resin into the desired 3D object. While this concept has been so far applied for photo-cross-linking and 3D printing, volumetric printing can be more broadly envisioned as a technique to spatially confine any light-triggered chemical reaction. In addition, as long as the printing resin is sufficiently transparent to the desired wavelength, the photoreaction could be conducted at any point in time post manufacture of a given object, in a noninvasive and biocompatible manner.

**Figure 1:**
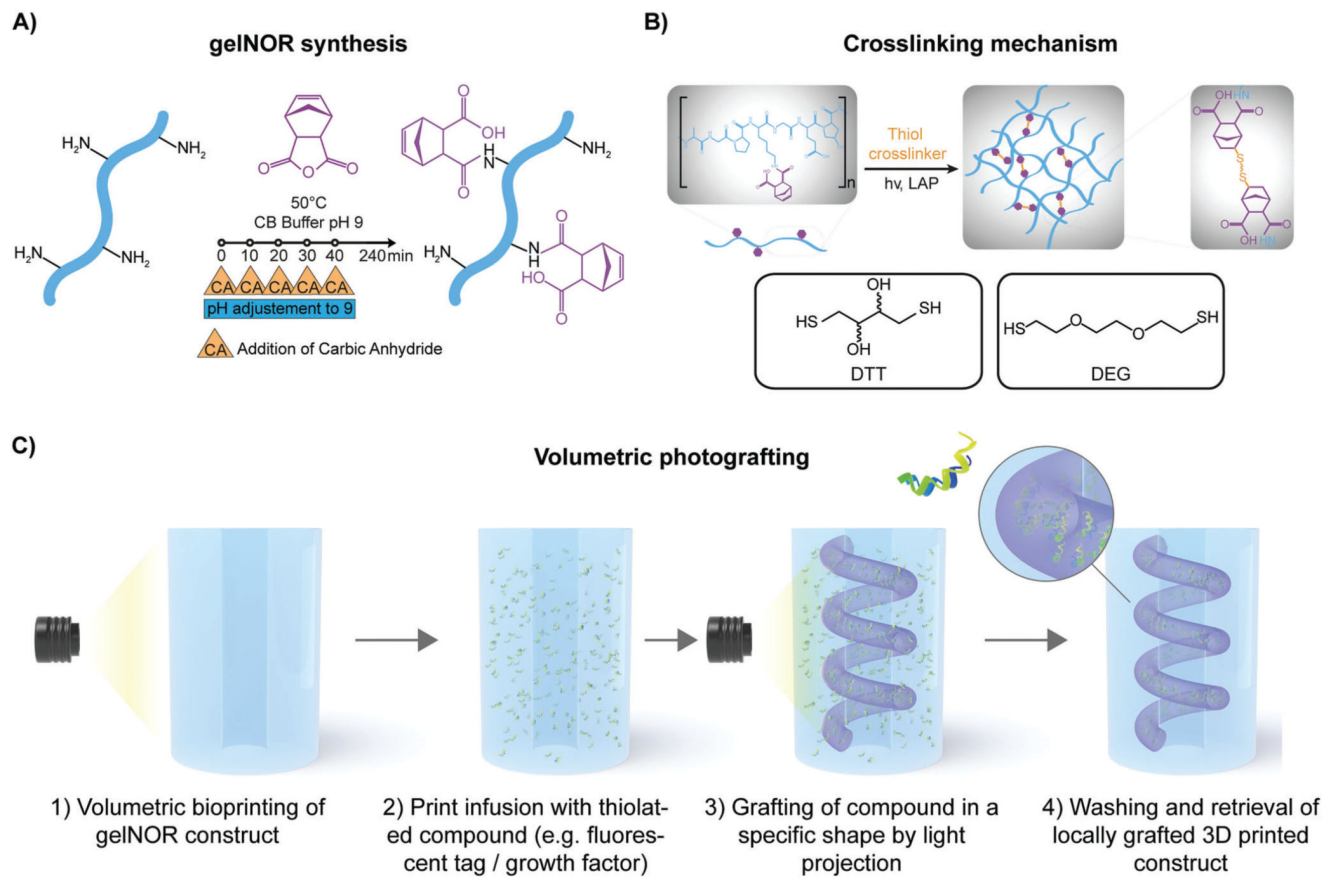
Overview of gelNOR synthesis and cross-linking reaction, and volumetric photografting process. A) Synthesis of gelNOR with addition of carbic anhydride at multiple time points. B) Schematic representation of the cross-linking mechanism of gelNOR with a dithiolated cross-linker, showing the two cross-linkers used in this study, DTT and DEG. C) Schematic representation of the volumetric photografting technique including volumetric printing, the infusion strategy with a thiolated compounds, and a second volumetric projection step for volumetric photografting of these compounds into complex structures within a gelNOR construct using covalent thiol–ene chemistry.

To demonstrate this concept, in this study, we selected gelatin as a base material due to its known biocompatibility and possibility to source it with low endotoxin content, which makes it potentially translatable for medical and pharmaceutical use.^[24]^ Moreover, gelatin allows for a broad array of chemical modifications, to accurately modulate its degradation profile and mechanical properties.^[25,26]^ As a platform material, we prepared and characterized a thiol–ene photo-cross-linkable norbornenemodified gelatin (gelNOR), which enables the generation of covalent hydrogel structures displaying complex geometries via volumetric printing. Thiol–ene click chemistry has gained increasing attention in the field of 3D printing and tissue engineering, as it yields hydrogels with highly homogenous network composition and mesh size. As the thiol–ene reaction progresses via a step-growth mechanism, the physical characteristics of the network can be reproducibly controlled by selecting the thiol-bearing cross-linker (length, molecular weight, number of reactive groups), network density, degree of functionalization (DoF), and the thiol-to-norbornene ratio.^[27]^ Given the accurate control over the cross-linking kinetics and reaction termination upon removal of light irradiation, it is also readily possible to contextually control the amount of unreacted norbornene groups, which remain available for secondary reactions (i.e., in this case, photografting) even after network percolation. Next, we subjected the volumetrically printed object to a second volumetric printing step in the presence of desired thiolated biomolecules of interest, a precise chemical editing can be performed locally at any point in time, decorating the hydrogel construct with 3D convoluted photopatterns (**Figure 1**). To ensure high spatial resolution during the photografting process, the interaction between tomographic light dose, initiator concentration, and inhibiting antioxidant compounds was thoroughly characterized. As a proof of concept of biological functionality, a hydrogel chip with a perfusable channel was produced and assessed for cell adhesion, spatioselective proliferation, and promoted self-assembly of endothelial cells seeded within the channel, in response to volumetrically grafted patterns of vascular endothelial growth factor (VEGF), a key chemokine in angiogenesis. Overall, this novel approach paves the way toward the production of future tissue culture scaffolds and biofabricated constructs that can be gradually modified to match the evolving, dynamic requirements of cells during tissue culture and maturation, thus offering a new toolbox toward the engineering of functional living tissues.

## 2 Results and Discussion

**Figure 2:**
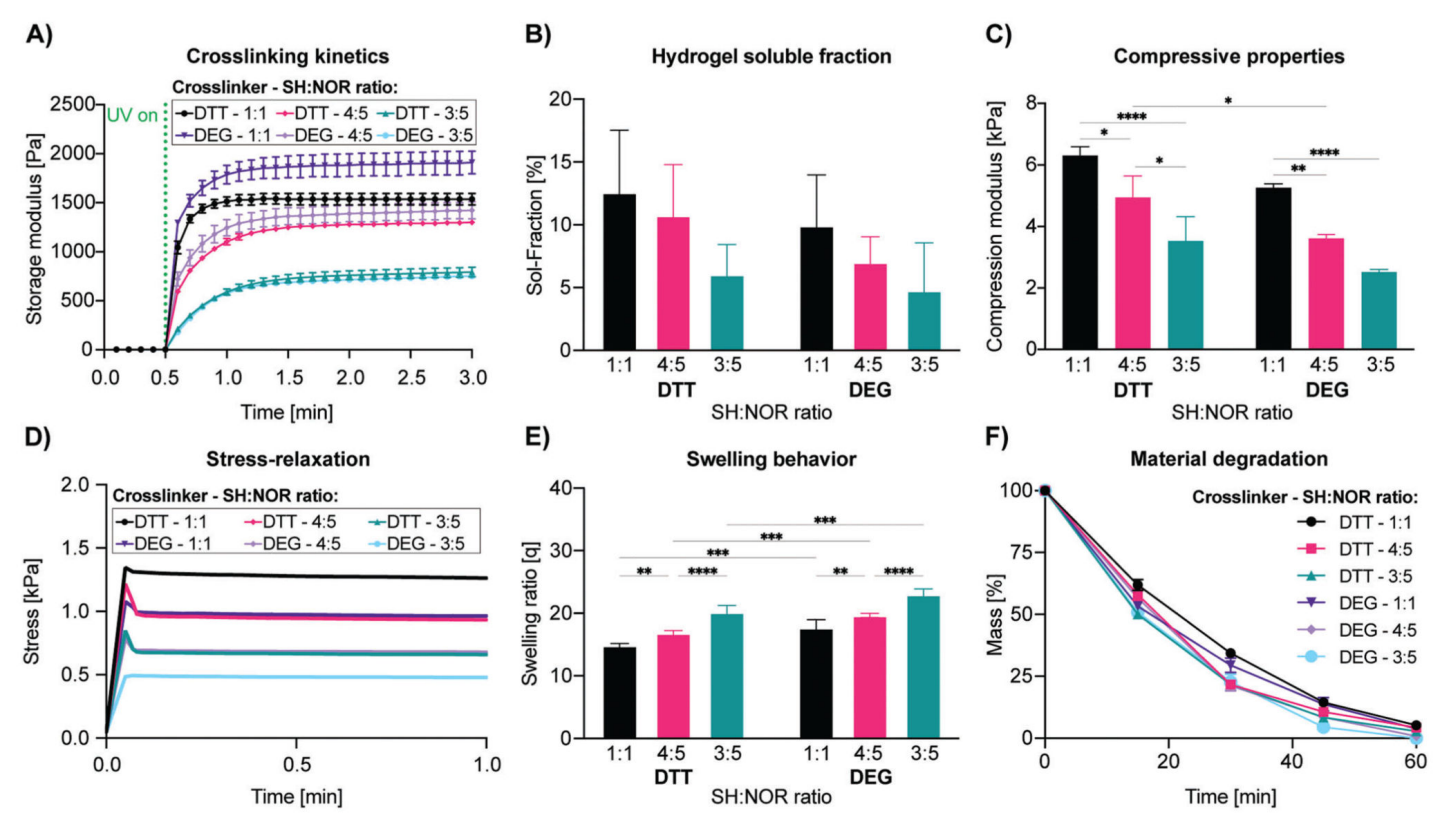
Mechanical and physical characterization of 5 w/v% gelNOR bioresins using different cross-linkers (DTT or DEG) at varying thiol–ene ratios (1:1, 4:5, or 3:5) keeping the LAP concentration consistent at 0.1 w/v%, showing A) photorheological time sweep measurements displaying the cross-linking kinetics (*n* = 3); B) soluble fraction of different gelNOR formulations (*n* = 5); C) compressive Young’s modulus (*n* = 3); D) stress-relaxation evolution graphs (*n* = 3); E) swelling behavior (*n* = 5); F) material degradation in collagenase solution at 37 °C (*n* = 3). * = *p* < 0.05, ** = *p* < 0.01, *** = *p* < 0.001, **** = *p* < 0.0001.

As a starting point, gelNOR was selected as it satisfies multiple requirements, namely: i) the compatibility with light-based 3D printing processes, to provide architectural control over 3D printed scaffolds of an arbitrary geometry, ii) the printing of structures with tunable mechanical properties across a broad range of stiffness relevant for tissue culture, via screening different dithiolated molecules, and iii) the ability to be used for photografting of any molecule bearing a free thiol group, such as those found in cysteine residues in native proteins. First, we screened an array of hydrogel formulations by varying cross-linker lengths and thiol–ene ratios, while keeping a constant 5 w/v% gelNOR concentration with 80% degree of norbornene functionalization, to maximize the amount of norbornene groups (Figure S1, Supporting Information). In addition, a relatively high degree of functionalization maximizes the number of reactive groups available for post-cross-linking during the volumetric photografting process. We thoroughly characterized a broad library of hydrogel formulations with tunable mechanical properties, by introducing two cross-linkers displaying different lengths: dithiothreitol (DTT) and a dithiolated diethyleneglycol (DEG), at different thiolto-norbornene ratios (**Figure 2**). To evaluate the general trend in the cross-linking kinetics of these hydrogel formulations, photorheology was performed (Figure 2A). We noticed that for all formulations, cross-linking of the hydrogels starts immediately upon the moment of light exposure and progresses with similar kinetics. This suggests that, in the range tested herein, the cross-linker length does not significantly influence the cross-linking kinetics of the hydrogel, which is in line with previous research.^[28]^ However, varying the thiol-to-norbornene ratios provided a clear difference in cross-linking kinetics between the samples, with 1:1 thiol-to-norbornene ratio yielding the fastest cross-linking kinetics (under 12 s to reach 80% of complete cross-linking). For hydrogels with a 4:5 thiol–ene ratio, the cross-linking was achieved in under 24 s and for the 3:5 thiol–ene ratio, cross-linking was achieved in under 36 s. The step-growth mechanism of gelNOR is known to provide rapid cross-linking, resulting from ring strain relief, especially when compared with hydrogels formed with a chain-growth mechanism, like gelatin methacryloyl (gelMA).^[28,29]^ Consequently, this allows to control the mesh size of the formed hydrogel network without greatly affecting the reaction kinetics, by changing the length of the cross-linker while maintaining constant the thiol–ene ratio. The soluble fraction (sol-fraction), which represents the amount of un-cross-linked polymer that washes out of the hydrogel network, of varying gelNOR formulations showed no significant difference for different cross-linkers or thiol-to-norbornene ratios (Figure 2B). Hydrogels formed with DTT as cross-linker displayed a sol-fraction of 12.58 ± 4.95%, 10.76 ± 4.03%, and 6.04 ± 2.39% for thiol–ene ratios 1:1, 4:5, and 3:5, respectively. Hydrogels formed with DEG as cross-linker provided a sol-fraction of 9.94 ± 4.04%, 7.00 ± 2.05%, and 4.78 ± 3.78% for the thiol–ene ratios 1:1, 4:5, and 3:5, respectively. All these measurements showed no significant difference. Dynamic mechanical analysis (DMA) was performed to determine the compressive modulus of the different hydrogel formulations (Figure 2C). Notably, a significant decrease in stiffness was observed as the thiol-to-norbornene ratios decreased in DTT samples (6.30 ± 0.29, 4.94 ± 0.70, and 3.53 ± 0.79 kPa for the thiol–ene ratios 1:1, 4:5, and 3:5) and between the 1:1 thiol–ene ratio (5.26 ± 0.13 kPa), the 4:5 ratio (3.62 ± 0.12 kPa), and the 3:5 ratio (2.52 ± 0.08 kPa) for DEG-cross-linked samples. This was to be expected since a 1:1 thiol–ene ratio would provide a maximal cross-linking of the polymer network and thus the stiffest gels, while at 4:5 and 3:5 thiol–ene ratios, there is an excess of norbornene groups that do not participate in the network. As for the effect of cross-linker length on the compressive properties of the hydrogels, a significant increase in stiffness (1.36-fold) was observed in DTT samples compared to DEG at the 4:5 thiol-to-norbornene ratio. This mechanical versatility supports previous data shown for this bioresin and demonstrates that the mechanical properties of gelNOR hydrogels can be easily tailored to specific needs by adjusting either the thiol–ene ratio, and/or the length of the thiol cross-linker.^[28–30]^ Considering the wide range of biomechanical requirements for culturing cells from different native tissues and organs, the mechanical versatility exhibited by gelNOR is of great interest to create stable, mechanically competent scaffolds for different tissue engineering applications.^[31]^ In terms of the stress relaxation response of the materials, all the formulations showed a predominantly elastic behavior, in line with the characteristics of covalent hydrogels, with minimal relaxation, and high retention of the peak stress upon application of a constant strain (Figure 2D and Figure S2 (Supporting Information)). The swelling ratio of the hydrogel formulations differed significantly, both for cross-linker length and thiol-to-norbornene ratio (Figure 2E). The swelling ratios for the hydrogels with DTT as cross-linker were 14.47 ± 0.62, 16.84 ± 0.42, and 20.18 ± 1.07 for thiol–ene ratios 1:1, 4:5, 3:5, respectively. For the hydrogels with DEG as cross-linker, we measured the swelling ratio to be 17.71 ± 1.28, 19.66 ± 0.33, and 23.02 ± 0.87 for the thiol–ene ratios 1:1, 4:5, and 3:5, respectively. These results show that the hydrogels with a 1:1 thiol–ene ratio have a significant difference with varying cross-linker lengths, where the longer DEG has a higher swelling ratio than the shorter DTT cross-linker, probably also because DEG has a more hydrophilic profile than DTT. Furthermore, the measurement showed a significant difference in varying thiol–ene ratios for the formulations with DTT as cross-linker, where we see that the lower the cross-linker density, the higher the swelling ratio. This indicates the higher cross-linking density to be effectively formed for the 1:1 thiol–ene ratio, as compared to the other thiol–ene ratios. To confirm that the tunability of mechanical and physical properties of the hydrogels did not hinder sample stability over time, the rate of degradation of the different gelNOR formulations was evaluated in the presence of low collagenase concentrations (Figure 2F). The results of this accelerated degradation test showed that all hydrogel formulations could be completely enzymatically degraded with a similar kinetics in a 60 min timeframe, therefore suggesting the potential for cultured cells to remodel the gelatin matrix.

**Figure 3:**
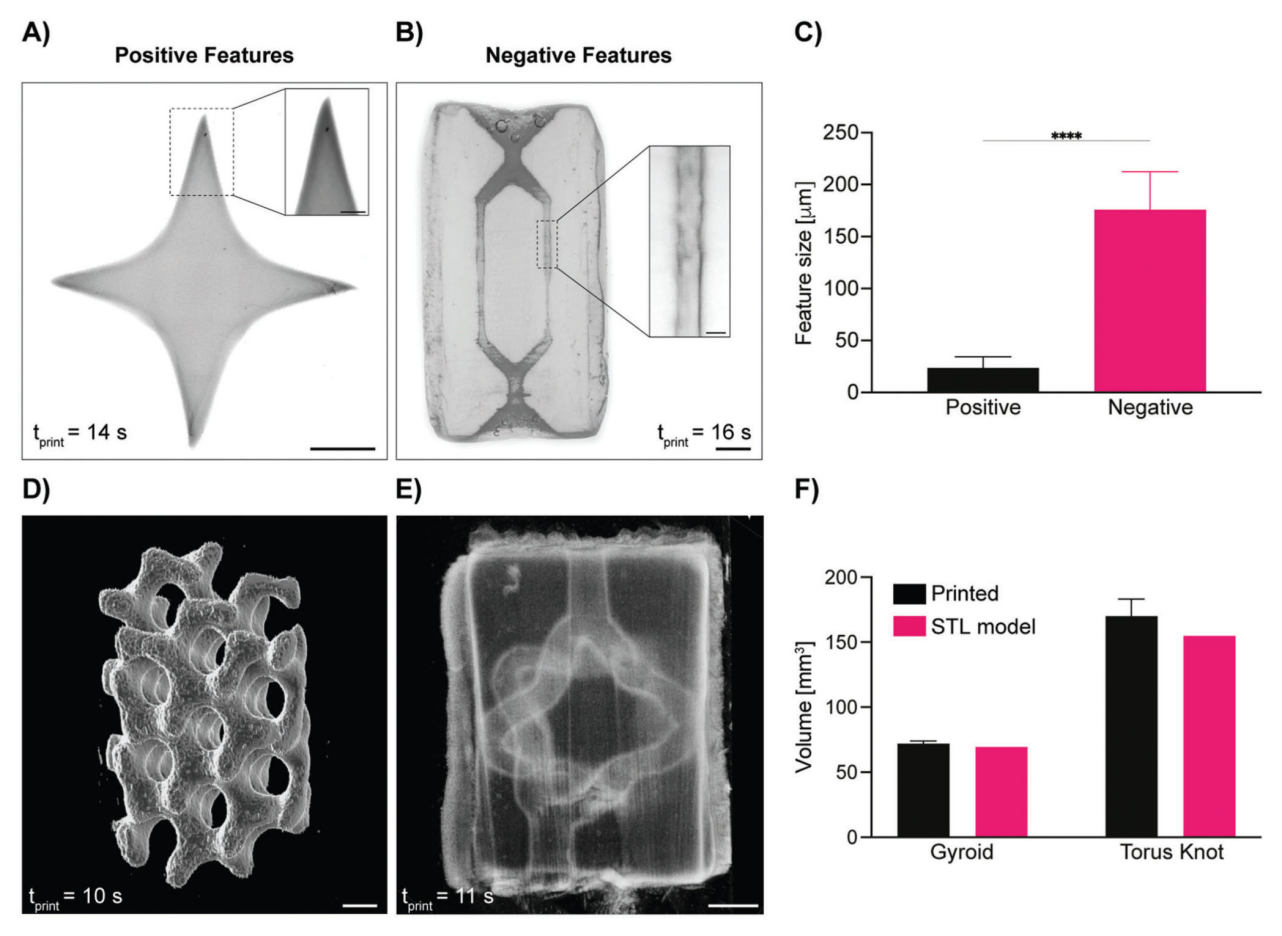
Volumetric printing resolution and accuracy using gelNOR bioresins. Stereomicroscopy images of A) positive and B) fully perfusable negative features achieved with the 1:1 DTT gelNOR formulation (scale bars = 1 mm; zoomed scale bar = 250 μm), and C) quantification of this maximum resolution (*n* = 3 independent samples, *n* = 10 technical replicates). Light-sheet 3D images of D) mathematically derived gyroid structure printed with gelNOR and E) hollow torus knot channel fabricated via VP using the 1:1 DTT gelNOR formulation (scale bars = 1 mm). F) Volume comparison between original STL file and the printed construct of the gyroid and torus knot structures (*n* = 3). **** = *p* < 0.0001.

Next, having available this set of photoresponsive hydrogels, the potential for shaping them into complex architectures through volumetric printing was investigated. For this, the formulation yielding 1:1 thiol–norbornene ratio and DTT as a cross-linker was used, as it was the one showing the highest mechanical stability and stiffness, thus allowing to maximize the ease of handling during printing and photografting. Light-based biofabrication technologies, such as stereolithography,^[32]^ digital light projection printing,^[33]^ and multiphoton lithography,^[34]^ enable printing at higher resolution (nanometers to tens of micrometers)^[35]^ and superior freedom of design compared to extrusion printing. In fact, being nozzle-free, light-based techniques sculpt photoresponsive materials, enabling the production of convoluted geometries recurrent in biological tissues (i.e., templates of vascular networks) that cannot be readily produced with conventional extrusion techniques. With the recent introduction of volumetric printing, such complex geometries can now also be produced with a resolution in the range of few tens of micrometers, while printing centimeter-sized objects in less than 20 s (**Figure 3**). To date, this technology has been applied to produce architecturally complex objects made of light-sensitive hydrogels,^[20,23,29]^ polymeric acrylic and thiol– ene resins,^[21,36]^ elastomers,^[37]^ nanoparticle-laden materials,^[38]^ and glass.^[39]^ In this study, we successfully achieved high printing resolutions with the selected gelNOR formulation of 23.68 ± 10.75 μm for positive features (e.g., spikes), and of 176.01 ± 36.34 μm, printing open, perfusable channels within a soft hydrogel matrix (Figure 3A,B). These findings show the highest printing resolution of positive features to date, and complement the high-speed, high-resolution printing of gelatin norbornene materials previously reported using this printing technique.^[29]^ Based on these printing conditions, more complex scaffolds were accurately resolved, from a mathematically derived gyroidal structure to torus-knot-shaped channels (Figure 3D,E). These highly convoluted structures were printed in less than 15 s, further underlining the ability of volumetric printing and of the gelNOR bioresin to rapidly and consistently produce architecturally complex, porous 3D structures. Printing accuracy was shown to be extremely high for both positive and negative feature constructs, showing no significant difference in volume between the digital model and the printed object itself (gyroid: 69.40 mm^3^ model vs 71.95 ± 2.11 mm^3^ print; torus knot channels: 154.73 mm^3^ model vs 169.85 ± 13.34 mm^3^ print) (Figure 3F).

**Figure 4:**
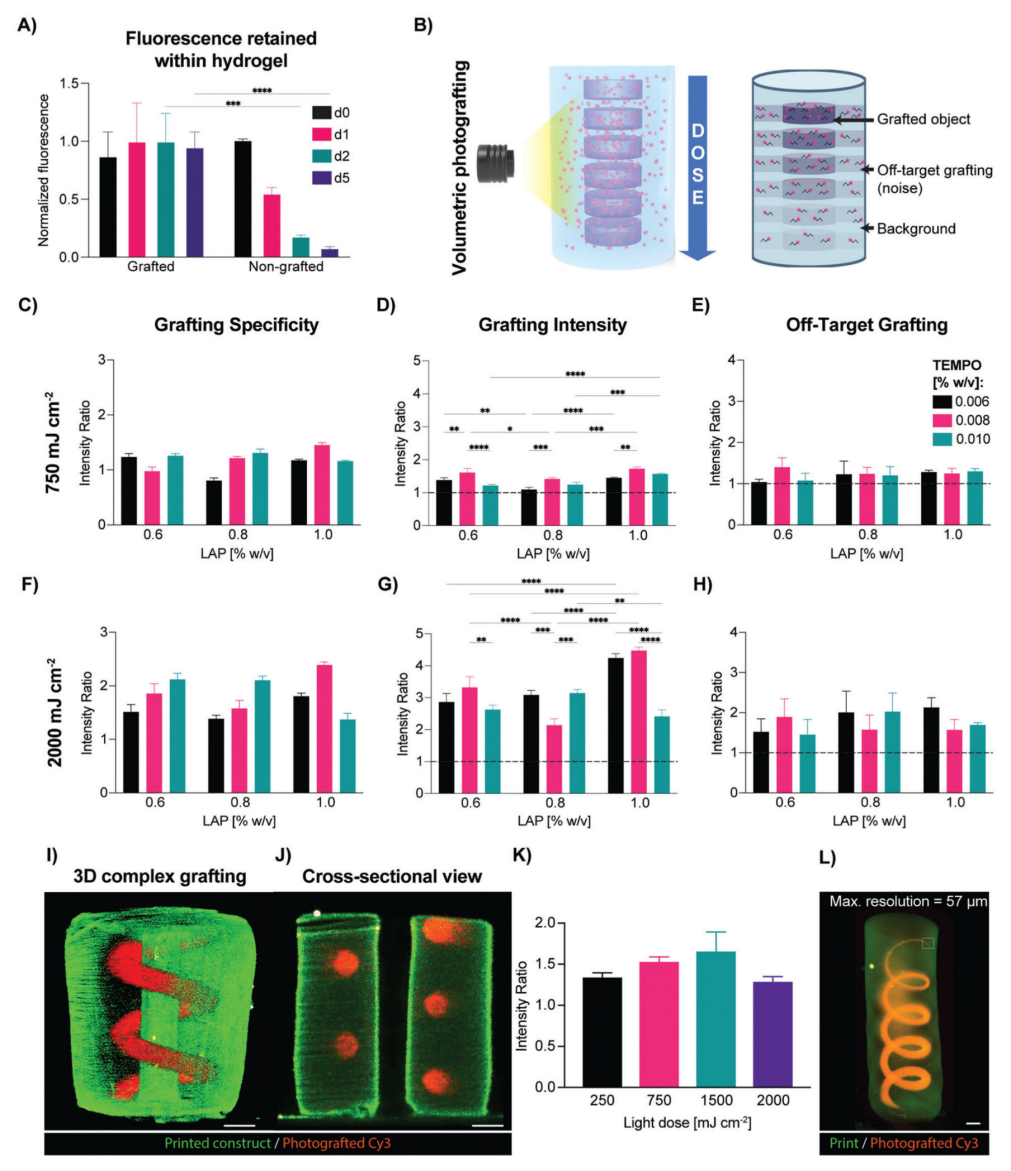
Optimization of the volumetric photografting of thiolated fluorescent compounds. A) Schematic representation of grafting optimization of gelNOR with Cy3–PEG–SH at different light doses, photoinitiator and cross-linking inhibitor concentrations. B) Normalized fluorescence intensity (λ = 580 nm) of grafted and nongrafted gelNOR hydrogels at different time points of washing with PBS (*n* = 3 technical replicates). C,F) Grafting specificity as ratio of intensity of the region of interest versus unwanted cross-linking. D,G) Grafting intensity as ratio between the intensity of the region of interest and the background (dotted line represents the baseline gelNOR autofluorescence). E,H) off-target grafting as ratio between the intensity of the unwanted photografted regions and the background of gelNOR (dotted line represents the baseline gelNOR autofluorescence). Samples were grafted at a dose of 750 or 2000 mJ cm^-2^ with different infusion mix concentrations (ranging from 0.6 to 1.0 w/v% LAP and from 0.006 to 0.010 w/v% TEMPO, with 0.06 w/v% Cy3–PEG–SH) (*n* = 3 technical replicates). I) 3D light-sheet reconstruction and J) cross-sectional view of a photografted Cy3–PEG–SH spiral inside a gelNOR construct with a central channel (scale bar = 1 mm). K) Grafting specificity of the photografted spiral at different light doses (250, 750, 1500, and 2000 mJ cm^-2^). L) 3D image of photografted coil structure of gradually decreasing width, starting at 5 mm, and measurement of maximum photografting resolution (scale bar = 1 mm). * = *p* < 0.05, ** = *p* < 0.01, *** = *p* < 0.001, **** = *p* < 0.0001.

Building on the high-resolution printability of the gelNOR resin, we then investigated the potential to functionalize the printed constructs by covalently cross-linking single thiol-bearing molecules on the gelatin backbone in a spatioselective fashion across centimeter-scale objects. During the volumetric printing process of hydrogels, light irradiation is on purpose prematurely stopped to avoid cross-linking of out-of-target regions of the build volume, which could lead to printing artefacts. Consequently, the hydrogel reaches enough network percolation to be considered stable, however the maximum cross-linking density is not achieved, and if necessary, can be reached only with a postcuring process.^[20,23]^ This feature is especially desirable for enabling secondary reactions postprinting, such is the case of photografting onto still available norbornene groups. As a first step, we thoroughly characterized the photografting process and how to modulate its accuracy, taking advantage of both the tomographic printing principle and the reactivity of the photoresin with thiols (**Figure 4**). For this purpose, we selected as a model molecule a fluorescent Cy3-tagged polyethylene glycol (PEG) chain functionalized with a single thiol moiety, which could be easily visualized and analyzed to determine photografting accuracy and intensity and exhibited stable fluorescence levels over time (Cy3–PEG–SH; 5 kDa; Figure S3, Supporting Information). As first step, it was first confirmed that the Cy3–PEG–SH compound could be covalently bound to the gelNOR network. To assess this, gelNOR cylinders were infused with the grafting cocktail (containing Cy3–PEG–SH and lithium phenyl-2,4,6-trimethylbenzoylphosphinate (LAP) as photoinitiator) and were either irradiated with light from the volumetric printer (2000 mJ cm^-2^), or left in the dark. As shown by fluorescence imaging, the photoexposed samples retained a stable level of fluorescence intensity over multiple days of incubation in phosphate-buffered saline (PBS). Conversely, the Cy3–PEG–SH rapidly diminished over time in non-photoexposed samples (Figure 4A). A quantitative assessment of the fluorescence measured in the PBS used to wash the hydrogels further corroborated this observation, displaying sixfold higher fluorescence signal in the eluates from the nonilluminated controls already after 1 day of incubation, showing a rapid release of the PEG probe as opposed to a stable incorporation facilitated by the volumetric printer (Figure S4, Supporting Information). Furthermore, quantitative analysis of the grafted and nongrafted samples demonstrated that for the grafting conditions selected in this experiment, the tethered monothiolated Cy3–PEG–SH was found to be in the range of 30.92 ± 2.06 μM concentration, and samples infused in the grafting cocktail but not photografted showed nondetectable dye concentrations (Figure S5, Supporting Information). Next, in order to ensure spatial control over the 3D patterns imparted during volumetric photografting, a thorough characterization of the reaction was performed. During the tomographic printing process, in fact, it is important to keep in mind that the whole hydrogel volume is exposed to light, by delivering an anisotropic, 3D dose distribution. With the aim to correctly confine the photografting reaction within the desired region dictated by the standard triangle language (STL) file, an optimal process would show high grafting specificity, which is a parameter measuring the contrast between in-target binding and off-target binding. As testing platform, gelNOR cylinders previously infused with a grafting cocktail were exposed to a series of disk-shaped tomographic projections using the volumetric printing setup and delivering to each disk a different light dose (750–2000 mJ cm^-2^), to screen grafting specificity, intensity, and degree of off-target grafting (Figure 4B and Figure S6 (Supporting Information)). It was initially observed that by simply adjusting the light dose delivered to the printed construct (750–2000 mJ cm^-2^) and photoinitiator concentration (0.6–1.0 w/v%), covalent photografting could be achieved, but the Cy3 dye was detected at nearly equal amounts everywhere across the light path traversing the hydrogel with low spatial specificity, likely due to the high reactivity of the gelNOR system (Figure S7, Supporting Information). We therefore hypothesized that slowing down the reaction kinetics by adding a free-radical inhibitor to the grafting cocktail could help minimize unwanted off-target events. In this study, we chose (2,2,6,6-tetramethylpiperidin-1-yl)oxidanyl (TEMPO) as inhibiting compound, since it has been previously used to enhance resolution in volumetric printing in combination with norbornene-based, nonhydrogel resins.^[40]^ At relatively high concentrations, TEMPO can act as a prooxidant and elicit cytotoxicity on bacterial and mammalian cells,^[41]^ however, this compound has been also proven to induce a protective effect for cells from oxidation-induced cell death,^[42]^ and to act as a reactive oxygen species (ROS) scavenger,^[43]^ when used in the safe concentration range also tested in our study (0.006–0.01 w/v%).^[41,44]^ At the lower average light dose tested (750–1250 mJ cm^-2^), regardless of the TEMPO concentration, low specificity ratios were still observed (0.804 ± 0.05–1.825 ± 0.08) and correlated with low grafting overall (both in- and off-target, Figure 4C–E and Figure S8 (Supporting Information)), the latter being indicative of limited reaction efficiency, in line with the inhibiting action of TEMPO. At higher light doses, instead, sufficient free radicals can be generated within the region of interest in the hydrogel, resulting in an improved contrast over the surrounding regions, which instead receive a lower dose as programmed by the tomographic algorithm and are therefore more affected by the presence of TEMPO (Figure 4F–H and Figure S8 (Supporting Information)). specifically, for the highest tested light dose (2000 mJ cm^-2^) and using the formulation consisting of 1.0 w/v% LAP and 0.008 w/v% TEMPO, grafting specificity of 2.388 ± 0.06 (2.1-fold higher than what was found without TEMPO) could be achieved, while also showing the highest in-target fluorescence intensity (4.473 ± 0.11 times higher than the background), and a low off-target intensity of 1.568 ± 0.26 (with 1 being the value of the native autofluorescence of the hydrogel). Altogether, these measurements showed that the grafting cocktail consisting of 1.0 w/v% LAP and 0.008 w/v% TEMPO allows for the most specific photografting to be achieved, while exhibiting dose-dependent intensity changes and greatly reducing off-target grafting. Having optimized the grafting cocktail to achieve highly specific spatial patterning of our fluorescent molecule, we explored the potential to photograft more complex architectures, and assessed the effect of light dose grafting specificity of the Cy3–PEG–SH compound. To assess this, a tubular spiral was grafted surrounding a central channel within a printed cylinder (Figure 4I,J). This structure was successfully patterned and visualized in 3D (Figure 4I,J). Previously, it has been shown in several studies employing the VP approach that different architectures, depending on their feature heterogeneity and size, require different light doses to be accurately resolved using this tomography-based approach.^[23]^ In the case of photografting of complex objects, a light dose sweep was performed to determine whether grafting specificity was in any way affected by light dose. We showed that, albeit the highest grafting specificity for the spiral pattern was found at 1500 mJ cm^-2^, there was no significant difference for the other tested selected light doses, which also managed to resolve the spiral structure. This could suggest that the optimal formulation of the infusion cocktail may yield a broad, robust window for grafting such convoluted geometry at high specificity (Figure 4K). On top of this large grafting window at different doses, our gelNOR photografting system also yielded high resolutions of the grafted objects within our volumetric prints. A grafted spiral starting at 5 mm in width (5.04 ± 0.08 mm grafted resolution) that gradually became thinner in width until reaching a resolution of 1 pixel in the digital file reached a fully grafted resolution of 57.20 ± 1.66 μm (Figure 4L). This high level of resolution could be of particular impact and interest to produce patterns of bioactive molecules mimicking the microscale organization of biochemical components found in native biological tissues even at a scale close to the size of a single mammalian cell.

**Figure 5:**
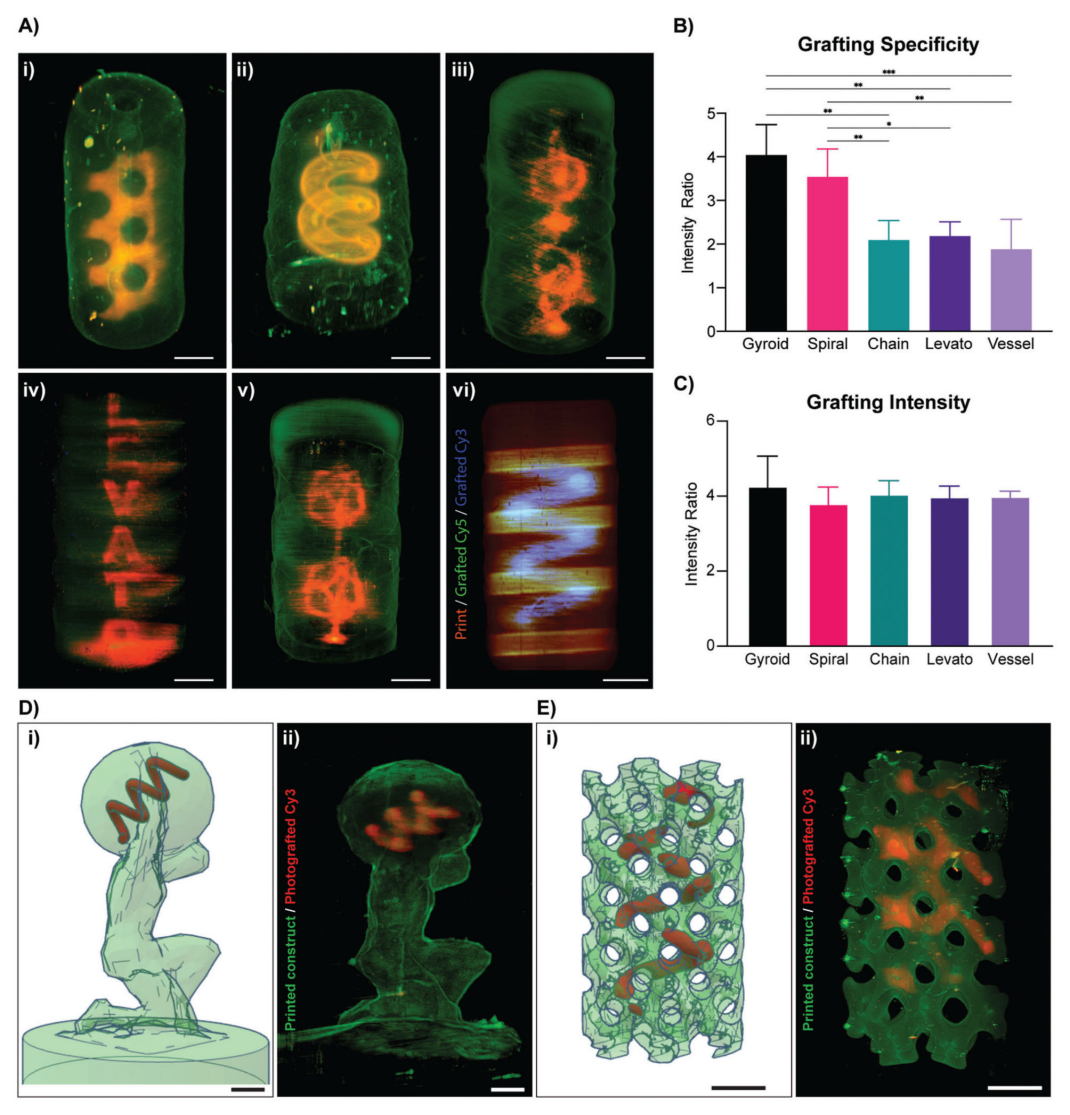
Volumetric photografting of complex structures. Photografting and analysis of geometries with different feature sizes and degrees of complexity. A) 3D image reconstructions of i) a mathematically derived gyroid, ii) a spiral, iii) interlocked chain model, iv) research lab name spelled vertically, v) a random interconnected vascular structure grafted with Cy3–PEG–SH onto cylindrical gelNOR volumetric prints, and vi) double dye grafting with Cy3–PEG–SH (spiral, blue) and Cy5–PEG–SH (disks, green) onto cylindrical gelNOR volumetric prints. B) Grafting specificity of complex grafting structures (*n* = 3 samples), and C) grafting intensity of complex grafting structures (*n* = 3 samples). D) i) STL file of an Atlas statue (green; CC BY-SA 3.0) volumetrically printed with gelNOR and a photografted Cy3–PEG–SH spiral (red). ii) Light-sheet 3D reconstruction of the printed gelNOR model (green) and the photografted spiral (red). E) i) STL file of a mathematically derived gyroid structure volumetrically printed with gelNOR (green) and a photografted Cy3–PEG–SH spiral (red). ii) Light-sheet 3D reconstruction of the printed gelNOR model (green) and the photografted spiral (red). Scale bars = 2 mm.

Having established a successful protocol for photografting structures at high resolution, a range of different structures with varying feature sizes and degrees of complexity were accurately grafted (**Figure 5** and Figure S9 (Supporting Information)). A highly tortuous, mathematically derived gyroidal structure surrounding a central hollow channel within a printed gelNOR cylinder (Figure 5A-i), a spiral structure surrounding a hollow channel (Figure 5A-ii), an interlocked chain structure with subunits in different axial orientations (Figure 5A-iii), the name of our research lab “Levato” spelled vertically along a gelNOR cylinder (Figure 5A-iv), and a random vessel structure (Figure 5A-v) were successfully grafted using the previously optimized grafting cocktail. Moreover, since most tissues present highly diverse types of proteins and growth factors critical for tissue function that are heterogeneously distributed along the same area, the possibility to graft multiple compounds in a spatially defined regions within the same printed object were also investigated. Here, a spiral shape was first grafted with Cy3–PEG–SH. Subsequently, another grafting process was performed, using a Cy5–PEG–SH, which was imprinted in the shape of vertically aligned cylinders (Figure 5A-vi). Grafting specificity of these complex geometries was measured for the gyroid (4.04 ± 0.70), spiral (3.54 ± 0.64), interlocked chain (2.09 ± 0.45), “Levato” (2.18 ± 0.33), and the random vessel (1.88 ± 0.69) (Figure 5B). Furthermore, grafting intensity of the complex geometries was measured for the gyroid (4.27 ± 0.80), spiral (3.80 ± 0.44), interlocked chain (4.05 ± 0.36), “Levato” (3.98 ± 0.29), and the random vessel (3.99 ± 0.14) (Figure 5C). The fact that both sets of values are within the same range as those observed in Figure 4 for simpler structures, further supports our previous observation that when using the optimized grafting cocktail, this process is extremely reproducible and as shown here, applicable to a wide range of architectures (Figure 5B). Variations in grafting specificity shown in Figure 5B are a phenomenon dependent on the tomographic reconstruction algorithm used for volumetric printing. As described in the previous literature,^[22]^ when delivering light doses from multiple angles following a Radon transform and filtered-backprojection-based algorithm, the exact light dose delivered in every voxel oscillates around the average light dose set by the user. As a result, regions at the borders of the construct, especially in presence of sharp corners, tend to receive slightly higher doses and react faster. Printing (and herein, grafting) artefacts caused by this phenomenon could be resolved with dedicated corrections of the tomographic algorithm, as previously shown.^[22]^ Despite this phenomenon, we demonstrate the possibility to accurately photograft complex patterns, even within more convoluted 3D printed structures, like an Atlas statue (Figure 5D) and a mathematically derived gyroid (Figure 5E). All in all, this fast method of grafting complex 3D patterns of several thiol-functionalized molecules can greatly increase the possibility of editing large hydrogel-based constructs in a spatiotemporally controlled fashion via sequential volumetric printing. Noteworthy to mention that thiol–ene chemistry is not the only possibility for photografting small molecules into a hydrogel system. In this study, thiol– ene chemistry was chosen since gelNOR is mechanically tunable and in many biological molecules there are cysteine residues capable of forming covalent networks through this thiol–ene chemistry. Other photochemistries could be studied for covalently grafting molecules to a hydrogel, i.e., dityrosine oxidation,^[45]^ photolysis of aromatic azides,^[46]^ or selectively cleaving areas in a gelatin hydrogel,^[28,47]^ which could further expand the library of functionalizing compounds that are usable with this volumetric photografting approach, to further enhance the biochemical profile of bioprinted scaffolds.

**Figure 6:**
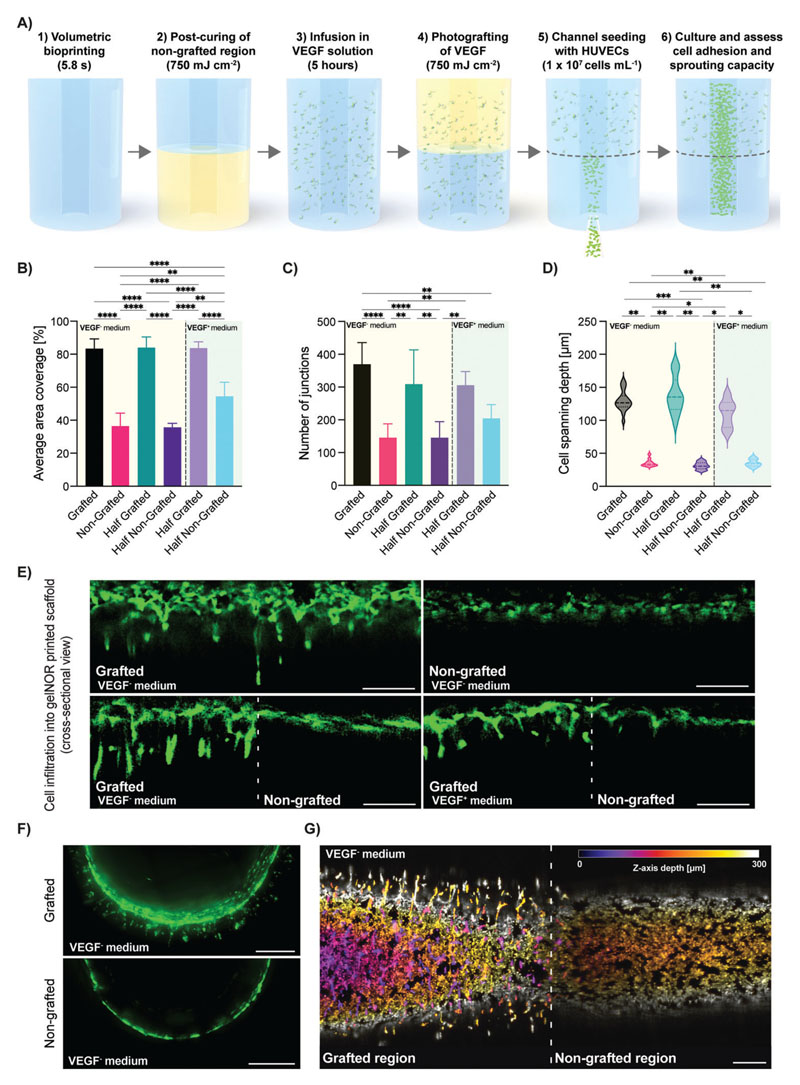
Photografting functional VEGF growth factor as proof-of-concept assay to guide endothelial cell adhesion, interconnectivity, and sprouting. A) Schematic diagram of the sterile process with which VEGF was photografted onto half of a vascular chip model consisting of a central channel within a gelNOR cylinder. B) Average area coverage and C) number of junctions of interconnected HUVEC clusters in the VEGF-grafted and nongrafted regions of the vascular chip cultured in VEGF-free and VEGF-supplemented medium after 3 days in culture (*n* = 3). D) Average HUVEC spanning depth from the channel wall into the VEGF-grafted and nongrafted regions of the vascular chip, cultured in VEGF-free and VEGF-supplemented medium after 3 days in culture (*n* = 3). E) Confocal images of horizontal cross-sections of the channel wall of the vascular chip seeded with HUVECs, showing various degrees of cell sprouting into the printed hydrogel. F) Confocal images of the vertical cross-section of the seeded vascular chip channel in grafted and nongrafted regions cultured in VEGF-free medium. G) Tile scan maximum projection image of a confocal z-stack, the vascular tube showing the boundary between the grafted and nongrafted regions (indicated by a dotted line). The color code indicates the *z*-axis depth, depicting how cells in all imaged layers can sprout in the surrounding hydrogel matrix. Scale bars = 250 μm. * = *p* < 0.05, ** = *p* < 0.01, *** = *p* < 0.001, **** = *p* < 0.0001.

Besides the tethering of fluorescent compounds for easy visualization and optimization of the photografting process, this approach can also be used to covalently attach proteins or growth factors within the printed structures for guiding cell fate with spatiotemporal control. While covalent grafting of biomolecules could have an effect on protein bioactivity, the use of norbornene moieties for thiol–ene photoclick chemistry has previously been shown to enable thiolated protein immobilization, with several growth factors showing maintained bioactivity post immobilization.^[48–52]^ As a proof of concept, we volumetrically grafted VEGF within a tissue-engineered macrochannel, aiming to improve the adhesion and sprouting capacity of human umbilical vein endothelial cells (HUVECs) within an uncoated lumen. VEGF expresses synergistic interactions with the integrin adhesion receptors guiding vessel growth and maturation, as well as endothelial cell survival by the regulation of antiapoptotic factor expression in these cells in vivo.^[53–55]^ This proangiogenic growth factor is routinely used in endothelial growth culture medium to selectively enhance vascularization in in vitro engineered models as well.^[56–60]^ VEGF has an uneven amount of cysteine residues and can be covalently coupled to a free norbornene onto the gelNOR network through the optimized photografting approach presented here. A volumetrically printed vascular chip, consisting of a central lumen of 1.5 mm in diameter, was fabricated to assess the effect of photografted VEGF on seeded HUVEC adhesion, interconnectivity, and sprouting capabilities (**Figure 6A**). Given the short half-life of recombinant VEGF protein, cell performance in the grafted and nongrafted regions of the printed samples was evaluated after 3 days to ensure the proangiogenic effects of the tethered growth factor were captured, in the presence of either VEGF-free (VEGF^-^) or VEGF-supplemented (VEGF^+^) medium. To ensure that only the effects of VEGF incorporation were analyzed, the nongrafted regions of the prints were postcured at the same light dose that was used to graft the VEGF, resulting in homogenous mechanical properties throughout the construct (Figure S10, Supporting Information). After 3 days of culture, clear differences in HUVEC adhesion and interconnectivity were observed in the VEGF-photografted regions cultured in both VEGF^+^ and VEGF^-^ culture media (Figure 6B,C and Figure S11 (Supporting Information)). In VEGF^-^, HUVEC adhesion in the grafted regions of the lumen was significantly enhanced compared to nongrafted areas, as shown by the increased average cell coverage in the grafted (84.06 ± 6.36%) versus nongrafted regions (35.72 ± 2.43%) (Figure 6B). In samples cultured with VEGF^+^ medium, the difference in the average HUVEC area coverage was less pronounced but showed significant differences between VEGF-grafted (83.71 ± 3.87%) and nongrafted (54.46 ± 8.57%) regions (Figure 6B) suggesting that in terms of cell adhesion to the hydrogel, the tethered VEGF provides a superior stimulation compared to free VEGF. Further, VEGF supplementation in the media did not significantly increase the coverage in the grafted regions of the construct, suggesting the absence of, or weak cumulative effects of the grafted and free soluble VEGF. Similarly, VEGF grafting had a significant effect on the cell interconnectivity, showing a higher number of intercluster junctions compared to regions lacking the covalently bound VEGF molecules in the absence of VEGF in the culture medium (309.00 ± 104.65 in VEGF-grafted region vs 145.40 ± 49.07 in nongrafted regions) (Figure 6C). These effects were conserved across VEGF^+^ and VEGF^-^ conditions. These observations suggest that the covalently tethered VEGF may provide a better support for HUVEC adhesion and growth compared to supplementation of soluble VEGF, at least in these initial stages of culture. The VEGF grafting could potentially be repeated over time to steer the vascular growth volumetrically printed constructs in real time in order to obtain more controlled multiscale vascular structures. Further, after only 3 days, the photografted VEGF facilitated HUVEC infiltration into the printed hydrogel, as shown by the significantly higher spanning depth of the cells from the inner edge of the lumen into the bulk hydrogel (130.77 ± 25.83 μm in VEGF-grafted regions vs 35.42 ± 7.73 μm in nongrafted regions) (Figure 6D,E). This observed cell infiltration was observed across the whole perimeter of the lumen (Figure 6F) and across the entire length of the printed channel (Figure 6G) in the grafted regions, while being completely absent in the non-grafted regions of the vascular chip model. These observations are encouraging, given that a proangiogenic effect is clearly seen in the VEGF-grafted regions of the lumen, where the growth factor acts as a chemoattractant capable not only of enhancing cell adhesion to an uncoated printed lumen, but also facilitates cell sprouting into the bulk hydrogel in the early days of culture. Since these effects are seen in both VEGF^-^ and VEGF^+^ medium, this strongly suggests that the covalently bound VEGF molecules retain sufficient bioactivity to steer the behavior of the seeded HUVECs. This grafting step could potentially be repeated over time to achieve different degrees of vascular growth volumetrically printed constructs and obtain more controlled multiscale vascular structures. Overall, this study proves the feasibility of grafting biologically functional compounds like growth factors, allowing these to maintain their bioactivity and guide cell fate with exceptional spatiotemporal control. Despite the demonstrated potential of this volumetric photografting technique, the infancy of this approach leaves room for future developments and exploration. To further boost the potential of the photografting process, a wider library of chemically editable bioresins suitable for VBP should be developed, and the stability and long-term functionality of different grafted compounds should be elucidated in more depth. Importantly, in terms of future perspectives, the biocompatibility of the grafting conditions toward cell viability and function should be assessed in long-term culture conditions to further evaluate translatability for potential regenerative medicine applications. Furthermore, with such future developments, the possibility to continuously edit the printed construct with different bioactive molecules during culture (i.e., to replenish the growth factor content over time, or to change its localization over time), can also be explored to more closely mimic certain developmental and tissue repair processes.

## 3 Conclusions

In this study, we demonstrated a new technological solution to create volumetric, 3D patterns of biological molecules within large, centimeter-scale hydrogels via tomographic printing and using visible light and bio-orthogonal thiol–ene chemistry. The selected material platform, gelNOR, was shown to possess highly controllable mechanical properties (through the adjustment of cross-linker length and resin DoF), and was shown to be printable via VBP, achieving a high printing resolution (20–30 μm for positive features). We demonstrated that these versatile gelNOR bioresins are suitable for the photografting of complex shapes onto volumetrically printed hydrogel constructs, as demonstrated by the controlled grafting of fluorescent dyes within gelNOR prints using tomographic projections, therefore allowing to both sculpt the architecture of the hydrogel and locally edit its chemical composition with high resolution (in the range of 50 μm). Through the extensive optimization of the grafting cocktail formulations (containing the thiolated compounds, cross-linking inhibitor TEMPO, and LAP photoinitiator) and the light dose delivered to the printed object, we achieved, for the first time, effective and precise photografting of both small dyes and large bioactive molecules, achieving micrometer-scale resolution of the grafted structures within centimeter-scale constructs while using a single-photon approach. As a proof of concept, we further applied this photografting principle to covalently tether the bioactive, proangiogenic growth factor VEGF to selectively guide and confine endothelial cell growth in the grafted, biofunctionalized areas. Improved cell adhesion and early formation of endothelial cell connections were observed preferentially in the biofunctionalized regions of the printed chip construct. Given that these observations match those of cells exposed to unbound VEGF molecules, this study indicates that the grafting process preserves bioactivity of the growth factors and opens the door for further characterization and tissue engineering applications. Overall, this work takes the first step in the characterization and development of smart materials that allow spatiotemporally precise biochemical editing. In combination with the ultrafast VBP technique, this photografting approach holds great promise to bring about the creation of biofabricated scaffolds that can better guide cell fate and behavior and therefore more closely mimic the complex biochemical environment of native tissues and organs.

## 4 Experimental Section

### Materials

Gelatin from porcine skin (type A, X-Pure low endotoxin content) was kindly provided by Rousselot Biomedical (Ghent, Belgium). Commercial grade gelNOR (type B, bovine hide, DoF 60%) was kindly provided by BIO INX BV (Zwijnaarde, Belgium). Cellulose dialysis membrane tubes (molecular weight cutoff = 12 kDa) were purchased from Sigma-Aldrich. LAP was purchased from Tokyo Chemical Industry (Tokyo, Japan). Cy3–PEG–SH and Cy5–PEG–SH (*M*w = 5 kDa) were purchased from Biopharma PEG (Watertown, USA). All other chemicals were obtained from Sigma-Aldrich unless stated otherwise.

### GelNOR Synthesis

Type A gelatin was dissolved in a carbonate– bicarbonate buffer (pH 9, 0.1 M concentration) to reach a 10 w/v% concentration. This solution was heated to 50 °C for the gelatin to dissolve and kept at this constant temperature throughout the synthesis. To reach a desired DoF, 0.2 g (1.2 mmol) of carbic anhydride (CA) per gram of gelatin was used in the reaction. The CA was added every 10 min for a total of 5 times starting at *t* = 0. After every addition of CA, the pH of the reaction was stabilized with 5 M NaOH to reach a pH of 9. After 240 min (DoF 80%) from the first addition of CA, the reaction was stopped by centrifuging the solution at 4000 rpm at room temperature for 5 min. Afterward, the pH was stabilized to 7.4 using 1 M HCl. To benchmark the custom-synthesized gelNOR, a commercial grade gelNOR kindly supplied by BIO INX BV (Zwijnaarde, Belgium) was used which exhibited comparable mechanical properties (Figure S12, Supporting Information) and grafting accuracy (Figure S13, Supporting Information) as the custom-synthesized hydrogel. The solution was diluted to reach a 5 w/v% concentration of gelatin and dialyzed against MilliQ water for 4 days at 4 °C. After the dialysis, the solution was further diluted with MilliQ to reach a final concentration of 2.5 w/v%. The solution was then heated to 50 °C and sterile filtered. Next, the solution was frozen at -80 °C, and lyophilized in a freeze dryer (Alpha 1-4 LSCbasic, Chris) to yield the dry product.

### Degree of Functionalization Quantification

2,4,6-trinitrobenzene sulfonic acid (TNBSA) assay was performed for the quantification of the amount of free amine groups present in the gelatin before and after functionalization. A glycine standard curve, to determine the amino group concentration, was prepared with concentrations of 0, 0.8, 8, 16, 32, 64 μg mL^-1^. Gelatin samples were dissolved in 1.6 mg mL^-1^ of 0.1 M NaHCO3 buffer. Subsequently, 0.5 mL of the sample was mixed with 0.5 mL of a 0.1 w/v% TNBSA solution in the buffer and incubated at 37 °C for 2 h. Next, the reaction was stopped by the addition of 0.25 mL of 1 M HCl and 0.5 mL of 10 w/v% sodium dodecyl sulfate. The absorbance of the samples was measured by a CLARIOstar Plus (BMG Labtech, Germany) plate reader at λ = 335 nm. The amount of free amines was calculated to be 0.3371 mmol per gram of gelatin, based on the TNBSA results (*n* > 5).

### Sample Preparation for Hydrogel Cross-Linking

Unless stated otherwise, all experiments were conducted using gelNOR hydrogel supplemented with the following components to achieve photo-cross-linking. GelNOR stock solutions were made in PBS at a 10 w/v% concentration. LAP stock solution was made in PBS at a 1 w/v% concentration. A stock solution of DTT or 2,2′-(ethylenedioxy)diethanethiol was prepared in PBS at a 100 mm concentration. To facilitate complete dissolution, all stock solutions were heated to 37 °C. Afterward, the stock solutions were mixed and diluted with PBS to reach a final concentration of 5 w/v% gelatin-based material, 0.1 w/v% LAP, and the tunable ratio of thiol cross-linker to norbornene as needed for each experiment (1:1, 4:5, or 3:5 thiol–ene ratio).

### Mechanical Analysis

GelNOR solutions from different aliquots of the same synthesis batch were casted in a cylindrical mold (6 mm diameter, 2 mm height), and cross-linked for 10 min (Cl-1000, Ultraviolet Cross-Linker, λ = 365 nm, *I* = 8 mW cm^-2^, UVP, USA). Samples were washed in PBS at 37 °C overnight to reach equilibrium swelling. To assess the compressive properties, the samples (*n* = 5) were subjected to a strain ramp at 20% min^-1^ strain rate until 30% deformation using a dynamic mechanical analyzer (DMA Q800, TA Instruments, The Netherlands). The compression modulus was calculated as the slope of the stress/strain curve in the 10– 15% linear strain range. To assess the viscoelastic properties, the samples (*n* = 5) were subjected to a strain recovery measurement at a constant 20% strain for 2 min and then left for recovery for 1 min, with a preload force of 0.0010 N. The elasticity index was calculated as the ratio between the recovered stress and the maximal stress under constant strain.

### Photorheology

Photorheology experiments on gelNOR precursor solutions to determine the cross-linking kinetics were assessed using a DHR2 rheometer (TA Instruments, The Netherlands). Time sweep experiments were performed at a frequency of 10.0 Hz, angular frequency of 62.83 rad s^-1^, with 5.0% constant strain at 21 °C (*n* = 3). A volume of 100 μL of gel was used with a gap size of 300 μm. A 20.0 mm parallel stainless steel electrically heated plate (EHP) was used as geometry. 30 s after the start of the measurement, the light source was activated (1200 mha, AOMEES, China, λ = 365 nm, intensity of 24 mW cm^-2^ for the remaining 2.5 min).

### Soluble Fraction and Swelling Ratio

The sol-fraction and swelling ratio experiment was performed according to a recent publication.^[29]^ Briefly, to assess sol-fraction of the gelNOR hydrogel formulation, cylindrical samples produced from different aliquots of the same synthesis batch (6 mm diameter, 3 mm height, *n* = 5) were weighed immediately after cross-linking for their initial mass. Next, samples were placed in PBS and placed in the incubator at 37 °C overnight. The next day, the hydrogel samples were weighed again, and their mass was measured as masswet,*t*0. Subsequently the hydrogels were lyophilized, and the dry mass (massdry,*t*0) was measured. The samples were stored in PBS again to ensure swelling of the dry gels and placed in the incubator at 37 °C overnight. The wet mass of the hydrogels was measured as masswet,*t*1. The samples were lyophilized, and the mass of the dry samples was measured as massdry,*t*1. The sol-fraction formula of the hydrogel formulations for analysis of the cross-linking properties of the gelNOR formulations was calculated with the following formula

(1) $$\text{Sol}-\text{fraction}\;\text{[\%]=}\frac{{\text{mass}}_{\text{dry,}t\text{0}}-{\text{mass}}_{\text{dry,}t\text{1}}}{{\text{mass}}_{\text{dry,}t\text{0}}}\times 100$$

The swelling ratio of the hydrogel formulations for analysis of the swelling behavior of the gelNOR hydrogel formulations was calculated with the following formula

(2) $$\text{Swelling}\;\text{ratio}\;(q)=\frac{{\text{mass}}_{\text{wet,}t\text{1}}}{{\text{mass}}_{\text{dry,}t\text{1}}}$$

### Enzymatic Degradation Assay

GelNOR hydrogels were swollen in PBS overnight and subsequently incubated in a 0.2 w/v% collagenase type II in Dulbecco’s modified Eagle medium (31966, Gibco, The Netherlands) supplemented with 10 v/v% heat-inactivated fetal bovine serum (FBS, Gibco, The Netherlands), and 1 v/v% penicillin and streptomycin (Life Technologies, The Netherlands) at 37 °C. Samples were removed from the enzymatic solution at different time points (15, 30, 45, and 60 min, *n* = 3 independent samples per time point). The mass of the hydrogel samples was measured and compared to the initial mass of the hydrogels before enzymatic incubation to determine the degradation rate of samples over time.

### Volumetric Printing

GelNOR solutions were dispensed into cylindrical borosilicate glass vials (*Ø* 10 mm), which were then loaded into a commercial volumetric 3D printer (Tomolite V1, Readily3D, Switzerland), equipped with a 405 nm laser, set to deliver an average light intensity of 11.98 mW cm^-2^ within the printing volume. Prior to printing, the samples were cooled to 4 °C to achieve physical gelation of the gelatin-based materials. Custom-designed STL files were loaded into the printer software (Apparite, Readily3D, Switzerland). After the printing process, the vials were heated to 37 °C and washed gently with 37 °C PBS to retrieve the prints. To ensure homogenous cross-linking, the sample was submerged in 0.1 w/v% solution of LAP in PBS and irradiated for 1 min in a UV oven.

### Volumetric Photografting

Printed constructs were subjected to a second printing step to induce spatioselective photografting. Samples were printed at equimolar amounts of thiol to norbornene at a 5 w/v% gelNOR concentration. Next, the printed samples were washed with PBS overnight, and infused with a fluorescent probe molecule, Cy3–PEG–SH (0.06 w/v%). To characterize the photografting reaction, several formulations of the infusion mix were prepared containing varying amounts of LAP (0.6, 0.8, or 1.0 w/v% concentration) and TEMPO (0, 0.006, 0.008, or 0.01 w/v% concentration), as inhibitor of the thiol–ene grafting reaction. The printed constructs were infused with the infusion mix at 4 °C for 2 h. Next, the gels were placed back into the printing vials with a small amount of gelatin (5 w/v% in PBS) to ensure thermal gelation and fixation of the construct inside the vial. The grafting process was performed in the printer, by loading STL files of the pattern to be grafted into the Apparite software, and performing a new tomographic light exposure step, to induce the 3D patterning of the fluorescent Cy3–PEG–SH in the programmed geometry. For the characterization of the volumetric grafting reaction, an array of vertically aligned cylindrical disks (3 mm diameter, 1 mm height) were grafted within a gelNOR cylinder (6 mm diameter, 20 mm height), with every disk exposed to a different dose (dose range: 250, 750, 1250, 1500, 1750, and 2000 mJ cm^-2^) (Figure 4A). The accuracy of the photografting process was assessed imaging cross-sections of these samples with a fluorescence microscope (Leica Microsystems, Germany), and the fluorescence intensity within the grafted regions of interest was compared to that of off-target areas. To assess the accuracy of photografting, 3 different ratios were calculated using the following formulas.

Grafting specificity formula for analysis of grafted GelNOR hydrogels with fluorescent dyes

(3) $$\text{Grafting}\;\text{specificity}\;\text{=}\frac{\text{Fluorescence}\;\text{of}\;\text{interest}\;\text{region}}{\text{Fluorescence}\;\text{of}\;\text{side}\;\text{bands}}$$

Grafting intensity formula for analysis of grafted GelNOR hydrogels with fluorescent dyes

(4) $$\text{Grafting}\;\text{intensity}\;\text{=}\frac{\text{Fluorescence}\;\text{of}\;\text{interest}\;\text{region}}{\text{Hydrogel}\;\text{autofluorescence}\;\text{(background)}}$$

Off-target grafting formula for analysis of grafted GelNOR hydrogels with fluorescent dyes

(5) $$\text{Off}\;\text{-}\;\text{target}\;\text{grafting}\;\text{=}\frac{\text{Fluorescence}\;\text{of}\;\text{side}\;\text{bands}}{\text{Hydrogel}\;\text{autofluorescence}\;\text{(background)}}$$

Using optimized grafting parameters, complex, arbitrary 3D patterns of the Cy3–PEG–SH were imparted within custom designed, 3D printed objects. Finally, the constructs were washed with PBS for a maximum of 5 days, until the un-cross-linked dye was completely removed from the gel. Subsequently, the photografted constructs were imaged with a light-sheet microscope. To demonstrate the possibility of grafting multiple molecules in a sequential fashion, a second grafting process was also performed using Cy5–PEG–SH as a fluorescent dye, using the same components of the grafting cocktail.

### Volumetric Grafting of VEGF and Cell Culture Assays

Cylindrical constructs with a perfusable channel spanning through the center of the construct were volumetrically printed as described above, and a proangiogenic growth factor was photografted on the bottom half of these constructs (*n* = 4 replicate samples, single HUVEC donor line). To ensure homogenous mechanical properties in the grafted and nongrafted regions, the bottom half (nongrafted) of the construct was postcured immediately after printing at the same light dose that was subsequently used during photografting (750 mJ cm^-2^). Samples were then washed and incubated overnight at 4 °C in an infusion mix of LAP (1 w/v%), TEMPO (0.008 w/v%), and recombinant human vascular endothelial growth factor (1000 ng mL^-1^; VEGF165, PeproTech). The volumetric photografting process was conducted as described above to deliver an average light dose of 750 mJ cm^-2^ to the top half of the construct and generate constructs with anisotropic VEGF patterning. The constructs were then washed at 37 °C for 5 h to remove excess, nongrafted VEGF. Green-fluorescent-protein (GFP)-tagged human umbilical vein endothelial cells (GFP–HUVECs, Angio-Proteomie, Boston, MA, USA, passage 5) were seeded into the channel within the printed construct at a concentration of 10^7^ cells mL^-1^. To ensure homogenous seeding through the round channel, the samples were placed in rectangular polydimethylsiloxane (PDMS) molds and rotated 90° every 15 min for the first hour of culture. Cell-seeded constructs were cultured in endothelial cell growth medium-2 (EGM-2) containing endothelial basal medium-2 + SingleQuots (except VEGF), 100 U mL^-1^–100 μg mL^-1^ PenStrep, and 10% heat-inactivated FBS. Samples were cultured at 37 °C and 5% CO_2_, medium was refreshed every day. To assess the effect of nongrafted VEGF, the full EGM-2 medium (including VEGF) was used for control samples. On day 3, fluorescent images of the GFP–HUVEC growing along the printed channels were acquired via confocal laser scanning microscopy (SPX8, Leica Microsystems, The Netherlands). The HUVEC area coverage and cell spanning depth (distance from inner side of the lumen to the outer edge of the lumen, or sprouting cells) were measured with Fiji,^[61]^ and junction numbers were analyzed using the vessel analysis software AngioTool.^[62]^

### Statistics

Results were reported as mean ± standard deviation. Statistical analysis was performed using GraphPad Prism 9 (GraphPad Software, USA). Comparisons between experimental groups were assessed via one or two-way analysis of variances (ANOVA), followed by post hoc Bonferroni correction to evaluate differences between groups. When normality could not be assumed, nonparametric tests were performed. differences were found to be significant when *p* < 0.05.

## Supplementary Material

Supplementary file

## Data Availability

The data that support the findings of this study are available from the corresponding author upon reasonable request.

## References

[1] Levato R, Jungst T, Scheuring RG, Blunk T, Groll J, Malda J (2020). Adv Mater.

[2] Groll J, Boland T, Blunk T, Burdick JA, Cho DW, Dalton PD, Derby B, Forgacs G, Li Q, Mironov VA, Moroni L (2016). Biofabrication.

[3] Wolf KJ, Weiss JD, Uzel SGM, Skylar-Scott MA, Lewis JA (2022). Cell Stem Cell.

[4] Gao B, Yang Q, Zhao X, Jin G, Ma Y, Xu F (2016). Trends Biotechnol.

[5] Mehrpouya M, Vahabi H, Janbaz S, Darafsheh A, Mazur TR, Ramakrishna S (2021). Polymer.

[6] Cao P, Tao L, Gong J, Wang T, Wang Q, Ju J, Zhang Y (2021). ACS Appl Polym Mater.

[7] Li G, Yan Q, Xia H, Zhao Y (2015). ACS Appl Mater Interfaces.

[8] Tognato R, Armiento AR, Bonfrate V, Levato R, Malda J, Alini M, Eglin D, Giancane G, Serra T (2019). Adv Funct Mater.

[9] López-Valdeolivas M, Liu D, Jan Broer D, Sánchez-Somolinos C, López-Valdeolivas M, Sánchez-Somolinos C, Liu D, Broer DJ, Sánchez-Somolinos CIBER in Bioengineering Biomaterials C (2018). Macromol Rapid Commun.

[10] Camarero-Espinosa S, Moroni L (2021). Nat Commun.

[11] Mulyasasmita W, Cai L, Dewi RE, Jha A, Ullmann SD, Luong RH, Huang NF, Heilshorn SC (2014). J Controlled Release.

[12] Madl CM, Heilshorn SC (2019). Chem Mater.

[13] Roppolo I, Frascella F, Gastaldi M, Castellino M, Ciubini B, Barolo C, Scaltrito L, Nicosia C, Zanetti M, Chiappone A (2019). Polym Chem.

[14] Cosola A, Chiappone A, Sangermano M (2022). Mol Syst Des Eng.

[15] Curley JL, Jennings SR, Moore MJ (2011). J Visualized Exp.

[16] Milind Gawade P, Shadish JA, Badeau BA, DeForest CA, Gawade PM, Shadish JA, Badeau BA, DeForest CA (2019). Adv Mater.

[17] Shadish JA, Benuska GM, DeForest CA (2019). Nat Mater.

[18] Batalov I, Stevens KR, DeForest CA (2021). Proc Natl Acad Sci USA.

[19] Broguiere N, Lüchtefeld I, Trachsel L, Mazunin D, Rizzo R, Bode JW, Lutolf MP, Zenobi-Wong M (2020). Adv Mater.

[20] Nuñez Bernal P, Delrot P, Loterie D, Li Y, Malda J, Moser C, Levato R, Bernal PN, Li Y, Malda J, Levato R (2019). Adv Mater.

[21] Kelly BE, Bhattacharya I, Heidari H, Shusteff M, Spadaccini CM, Taylor HK (2019). Science.

[22] Loterie D, Delrot P, Moser C (2020). Nat Commun.

[23] Bernal PN, Bouwmeester M, Madrid-Wolff J, Falandt M, Florczak S, Rodriguez NG, Li Y, Größbacher G, Samsom RA, van Wolferen M, van der Laan LJW (2022). Adv Mater.

[24] Groen WMGAC, Utomo L, Castilho M, Gawlitta D, Malda J, van Weeren PR, Levato RM, Korthagen N (2020). Int J Mol Sci.

[25] van Hoorick J, Tytgat L, Dobos A, Ottevaere H, van Erps J, Thienpont H, Ovsianikov A, Dubruel P, van Vlierberghe S (2019). Acta Biomater.

[26] Buie T, McCune J, Cosgriff-Hernandez E (2020). Trends Biotechnol.

[27] Bertlein S, Brown G, Lim KS, Jungst T, Boeck T, Blunk T, Tessmar J, Hooper GJ, Woodfield TBF, Groll J (2017). Adv Mater.

[28] van Hoorick J, Dobos A, Markovic M, Gheysens T, van Damme L, Gruber P, Tytgat L, van Erps J, Thienpont H, Dubruel P, Ovsianikov A (2020). Biofabrication.

[29] Rizzo R, Ruetsche D, Liu H, Zenobi-Wong M, Rizzo R, Ruetsche D, Liu H, Zenobi-Wong M (2021). Adv Mater.

[30] van Hoorick J, Gruber P, Markovic M, Rollot M, Graulus GJ, Vagenende M, Tromayer M, van Erps J, Thienpont H, Martins JC, Baudis S (2018). Macromol Rapid Commun.

[31] Cox TR, Erler JT (2011). Dis Models Mech.

[32] Karakurt I, Aydog˘du A, Çıkrıkcı S, Orozco J, Lin L (2020). Int J Pharm.

[33] Levato R, Lim KS, Li W, Asua AU, Peña LB, Wang M, Falandt M, Bernal PN, Gawlitta D, Zhang YS, Woodfield TBF (2021). Mater Today Bio.

[34] Dobos A, Gantner F, Markovic M, van Hoorick J, Tytgat L, van Vlierberghe S, Ovsianikov A (2021). Biofabrication.

[35] Zandrini T, Florczak S, Levato R, Ovsianikov A (2022). Trends Biotechnol.

[36] Cook CC, Fong EJ, Schwartz JJ, Porcincula DH, Kaczmarek AC, Oakdale JS, Moran BD, Champley KM, Rackson CM, Muralidharan A, McLeod RR (2020). Adv Mater.

[37] Loterie D, Delrot P, Moser C (2018).

[38] Madrid-Wolff J, Boniface A, Loterie D, Delrot P, Moser C (2022). Adv Sci.

[39] Toombs JT, Luitz M, Cook CC, Jenne S, Li CC, Rapp BE, Kotz-Helmer F, Taylor HK (2022). Science.

[40] Cook CC, Fong EJ, Schwartz JJ, Porcincula DH, Kaczmarek AC, Oakdale JS, Moran BD, Champley KM, Rackson CM, Muralidharan A, McLeod RR (2020). Adv Mater.

[41] Guo X, Mittelstaedt RA, Guo L, Shaddock JG, Heflich RH, Bigger AH, Moore MM, Mei N (2013). Toxicol In Vitro.

[42] Pattison DI, Lam M, Shinde SS, Anderson RF, Davies MJ (2012). Free Radical Biol Med.

[43] Li J, Zhang J, Chen Y, Kawazoe N, Chen G (2017). ACS Appl Mater Interfaces.

[44] Guo X, Seo JE, Bryce SM, Tan JA, Wu Q, Dial SL, Moore MM, Mei N (2018). Toxicol Sci.

[45] Lim KS, Abinzano F, Bernal PN, Albillos Sanchez A, AtienzaRoca P, Otto IA, Peiffer QC, Matsusaki M, Woodfield TBF, Malda J, Levato R (2020). Adv Healthcare Mater.

[46] Ovsianikov A, Li Z, Ajami A, Torgersen J, Husinsky W, Stampfl J, Liska R (2012). Appl Phys A: Mater Sci Process.

[47] Sayer S, Zandrini T, Markovic M, van Hoorick J, van Vlierberghe S, Baudis S, Holnthoner W, Ovsianikov A (2022). Sci Rep.

[48] McCall JD, Anseth KS (2012). Biomacromolecules.

[49] Sridhar Bv, Doyle NR, Randolph MA, Anseth KS (2014). J Biomed Mater Res., Part A.

[50] Wang G, Yuan N, Li N, Wei Q, Qian Y, Zhang J, Qin M, Wang Y, Dong S (2022). ACS Appl Mater Interfaces.

[51] Sridhar Bv, Brock JL, Silver JS, Leight JL, Randolph MA, Anseth KS (2015). Adv Healthcare Mater.

[52] Weaver JD, Headen DM, Aquart J, Johnson CT, Shea LD, Shirwan H, García AJ (2017). Sci Adv.

[53] Ferrara N, Gerber HP, LeCouter J (2003). Nat Med.

[54] An Y, Liu WJ, Xue P, Ma Y, Zhang LQ, Zhu B, Qi M, Li LY, Zhang YJ, Wang QT, Jin Y (2018). Cell Death Dis.

[55] Ge Q, Zhang H, Hou J, Wan L, Cheng W, Wang X, Dong D, Chen C, Xia J, Guo J, Chen X (2018). Mol Med Rep.

[56] Poldervaart MT, Gremmels H, van Deventer K, Fledderus JO, Öner FC, Verhaar MC, Dhert WJA, Alblas J (2014). J Controlled Release.

[57] Takei T, Sakai S, Ono T, Ijima H, Kawakami K (2006). Biotechnol Bioeng.

[58] Wang X, Phan DTT, Sobrino A, George SC, Hughes CCW, Lee AP (2016). Lab Chip.

[59] Chen RR, Silva EA, Yuen WW, Mooney DJ (2007). Pharm Res.

[60] Freeman FE, Pitacco P, van Dommelen LHA, Nulty J, Browe DC, Shin JY, Alsberg E, Kelly DJ (2020). Sci Adv.

[61] Schindelin J, Arganda-Carreras I, Frise E, Kaynig V, Longair M, Pietzsch T, Preibisch S, Rueden C, Saalfeld S, Schmid B, Tinevez JY (2012). Nat Methods.

[62] Zudaire E, Gambardella L, Kurcz C, Vermeren S (2011). PLoS One.

