# Supplementary material for: Spatial-Selective Volumetric 4D Printing and Single-Photon Grafting of Biomolecules within Centimeter-Scale Hydrogels via Tomographic Manufacturing
Source: Adv Mater Technol. Author manuscript; Available in PMC 2023 Oct 6. (PMC7615165; doi:10.1002/admt.202300026)
Supplement: Supplementary file [file EMS188654-supplement-Supplementary_file.pdf]

ADVANCED MATERIALS TECHNOLOGIES

Supporting Information

for *Adv. Mater. Technol.*, DOI 10.1002/admt.202300026

Spatial-Selective Volumetric 4D Printing and Single-Photon Grafting of Biomolecules within Centimeter-Scale Hydrogels via Tomographic Manufacturing

*Marc Falandt, Paulina Nuñez Bernal, Oksana Dudaryeva, Sammy Florczak, Gabriel Größbacher, Matthias Schweiger, Alessia Longoni, Coralie Greant, Marisa Assunção, Olaf Nijssen, Sandra van Vlierberghe, Jos Malda, Tina Vermonden and Riccardo Levato**

Supplementary Information

Spatial-selective Volumetric 4D Printing and Single-photon Grafting of Biomolecules within Centimeter-scale Hydrogels via Tomographic Manufacturing

*Marc Falandt, Paulina Nuñez Bernal, Oksana Dudaryeva, Sammy Florczak, Gabriel Groessbacher, Matthias Schweiger, Alessia Longoni, Coralie Greant, Marisa Assunção, Olaf Nijssen, Sandra van Vlierberghe\, Jos Malda, Tina Vermonden, Riccardo Levato**

M. Falandt, M. Schweiger, O. Nijssen, J. Malda, R. Levato. Department of Clinical Sciences, Faculty of Veterinary Medicine, Utrecht University, 3584CT, Utrecht, the Netherlands. Email: r.levato@uu.nl

P.N. Bernal, O. Dudaryeva, S. Florczak, G. Groessbacher, A. Longoni, M. Assunção, J. Malda, R. Levato. Departement of Orthopaedics, University Medical Center Utrecht, Utrecht University, 3584CX, Utrecht, The Netherlands.

C. Greant, S. Van Vlierberghe. Polymer Chemistry & Biomaterials Group, Centre of Macromolecular Chemistry, Department of Organic & Macromolecular Chemistry, Faculty of Sciences, Ghent University, 9000, Ghent, Belgium

C. Greant, S. Van Vlierberghe. BIO INX BV, Technologiepark-Zwijnaarde 66, 9052, Ghent, Belgium

T. Vermonden. Department of Pharmaceutical Sciences, Faculty of Science, Utrecht University, 3584CG, Utrecht, the Netherlands

*Correspondence to: r.levato@uu.nl

Supplementary Figures

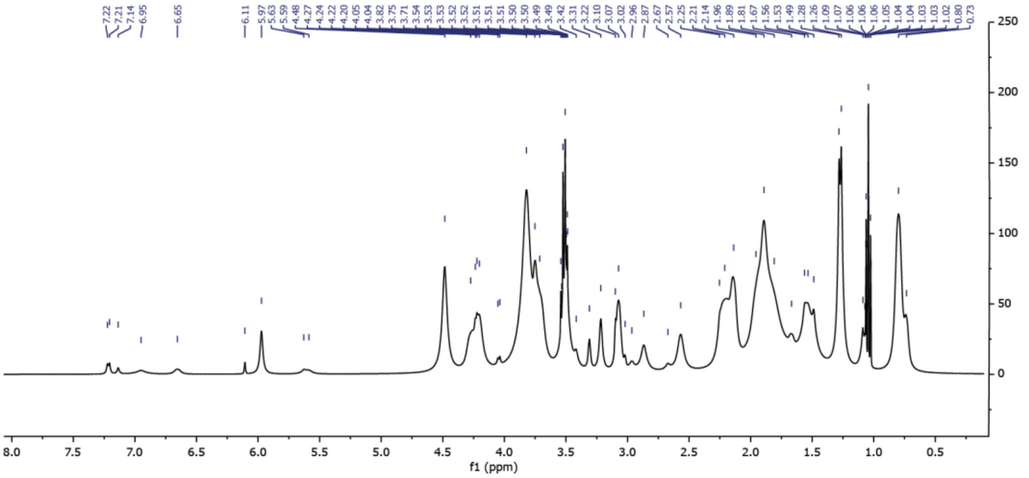

Supplementary Figure S1: ^{1}H-NMR spectrum of gelNOR in D_2O. ^{1}H NMR (400 MHz, D_2O) δ = 5.97 (s, 1H), 4.48 (s, 3H), 4.30 – 4.18 (m, 3H), 3.82 (s, 10H), 3.75 (s, 0H), 3.71 (s, 2H), 3.52 (dd, *J* = 7.4, 3.8 Hz, 1H), 3.50 (s, 3H), 3.22 (s, 1H), 3.07 (s, 2H), 2.87 (s, 1H), 2.57 (s, 1H), 2.21 (s, 3H), 2.14 (s, 2H), 1.96 (s, 1H), 1.89 (s, 8H), 1.81 (s, 6H), 1.58 – 1.51 (m, 4H), 1.49 (s, 1H), 1.27 (d, *J* = 7.5 Hz, 5H), 1.11 – 1.00 (m, 2H), 0.80 (s, 5H), 0.73 (s, 1H).

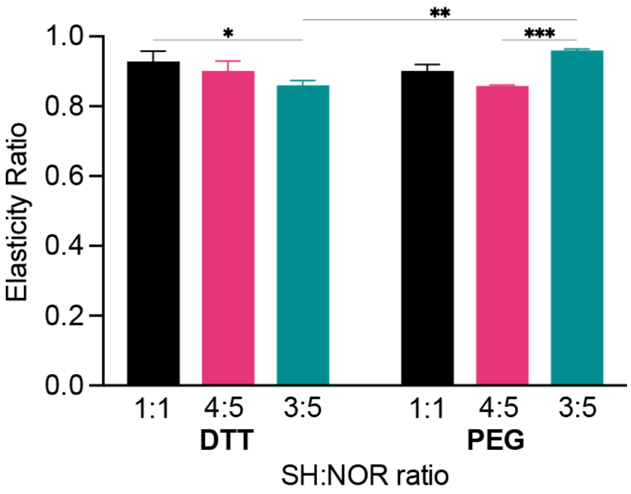

Supplementary Figure S2: Elasticity ratio of gelNOR hydrogels employing crosslinkers of different lengths and different thiol-to-norbornene molar ratios as measured by DMA as a result of stress-relaxation measurements (n = 3). * = p < 0.05, ** = p < 0.01, *** = p < 0.001.

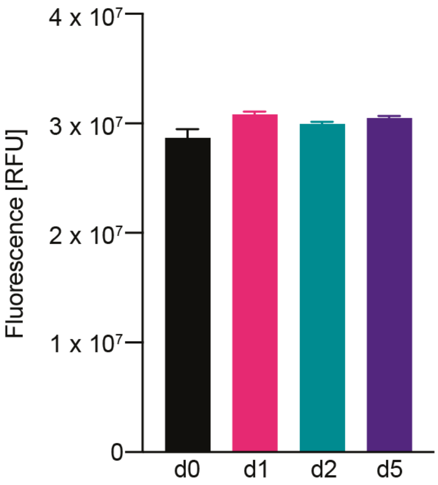

Supplementary Figure S3: Fluorescent intensity of the infusion mix after infusing of the hydrogel samples at different time points. Showing no significant decrease in fluorescence over time (n = 3).

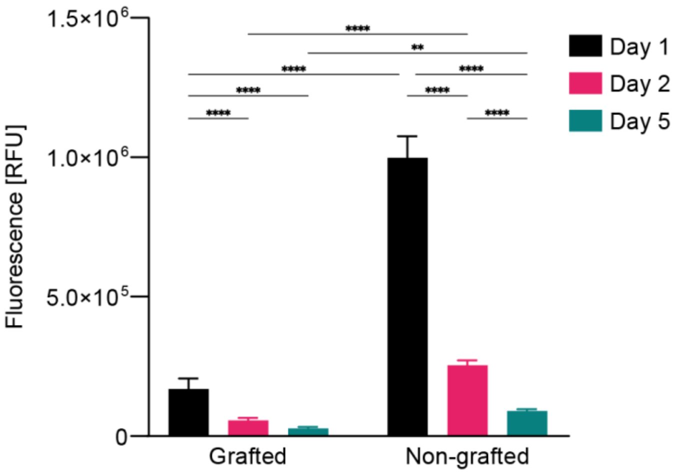

Supplementary Figure S4: Fluorescent intensity of washing water of both grafted and non-grafted gelNOR hydrogels at different time points (n = 5). ** = p < 0.01, *** = p < 0.001, **** = p < 0.0001.

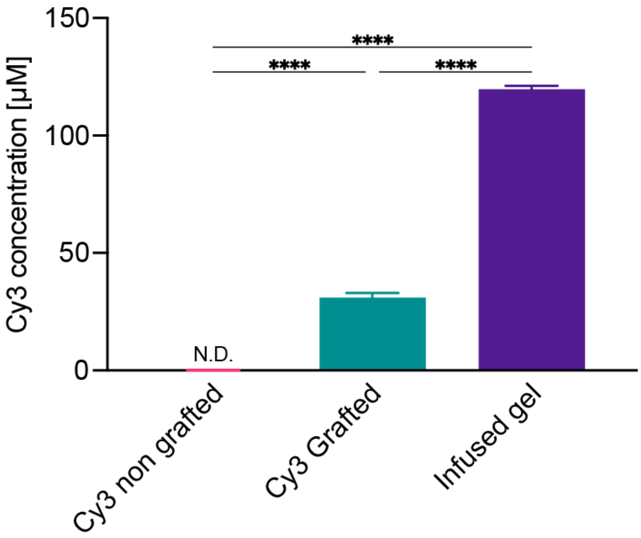

Supplementary Figure S5. Cy3-PEG-SH concentration quantification of non-infused, non-grafted and grafted gelNOR hydrogels after washing. The hydrogel samples were enzymatically degraded using 0.2 w/v% collagenase, after which the fluorescent intensity was measured and the concentration of dye inside the hydrogel was calculated (n = 3). N.D. = Not Detected. **** = p < 0.0001.

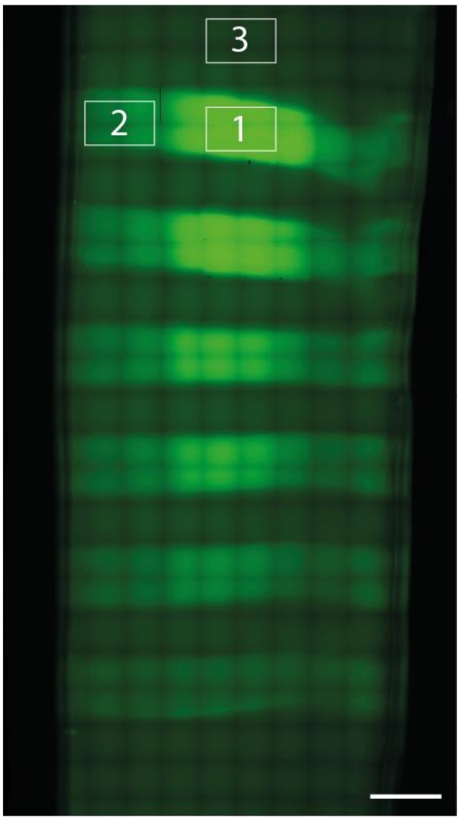

Supplementary Figure S6: Fluorescence image of gelNOR cylinder as used for grafting optimization used in **Figure 4** to optimize photografting, explaining how the ratios were measured. Area 1 is used to measure the intensity of the intended photografting region. Area 2 is used to measure intensity off-target grafting of the unwanted regions. Area 3 is used to measure the intensity of the background. From these values, the grafting specificity (ratio of area 1 vs. area 2), grafting intensity (ratio of area 1 vs. area 3), and off-target grafting (ratio of area 2 vs. area 3) was measured. This image is from a gelNOR hydrogel with a formulation consisting of 1.0 w/v% LAP, 0.006 w/v% TEMPO, and 0.06 w/v% Cy3-PEG-SH. Scalebar = 1 mm.

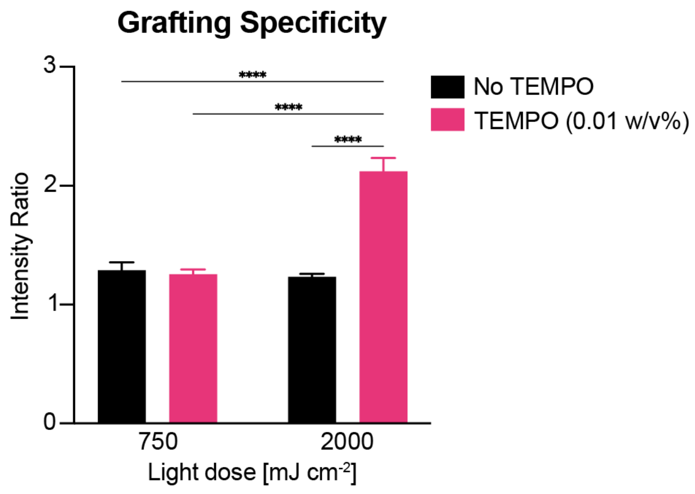

Supplementary Figure S7: Grafting specificity of Cy3-PEG-SH on gelNOR hydrogels without radical scavenger TEMPO and with 0.010 w/v% TEMPO included in the grafting cocktail, with a stable photoinitiator concentration (0.6 w/v% LAP) and at two volumetric printing light doses (750 and 2000 mJ/cm^2) (n = 3). **** = $p < 0.0001$

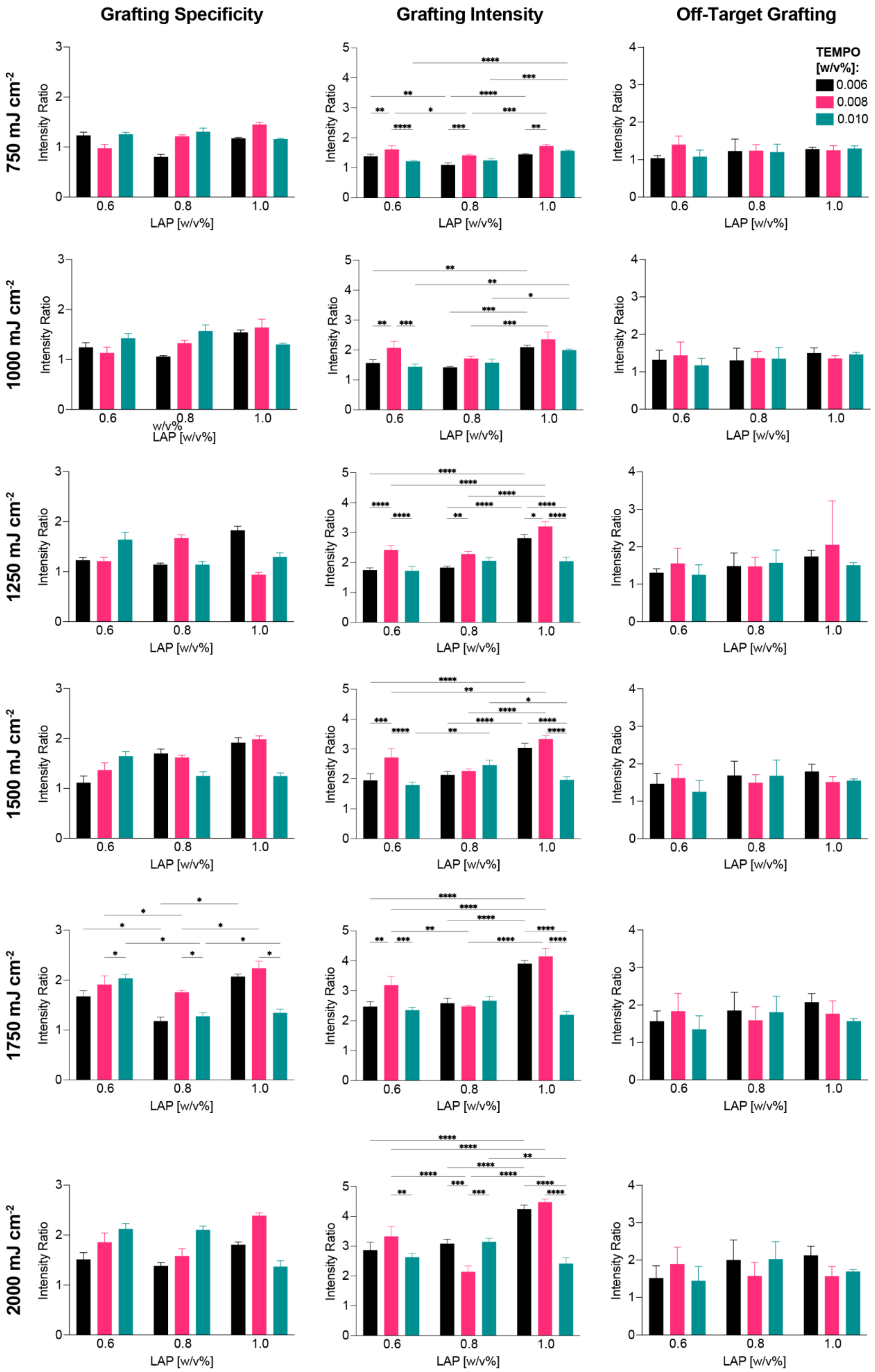

Grafting Specificity
Grafting Intensity
Off-Target Grafting
TEMPO [w/v%]:
0.006
0.008
0.010
750 mJ cm-2
1000 mJ cm-2
1250 mJ cm-2
1500 mJ cm-2
1750 mJ cm-2
2000 mJ cm-2
Intensity Ratio
LAP [w/v%]
0.6
0.8
1.0
w/v%

Supplementary Figure S8: Full grafting optimization (specificity, intensity and off-target grafting) of Cy3-PEG-SH on gelNOR hydrogels at different light doses (750 – 2000 mJ/cm^2), photoinitiator (0.6 - 1.0 w/v%) and crosslinking inhibitor concentrations (0.006 - 0.010 w/v%) (n = 3). * = $p < 0.05$, ** = $p < 0.01$, *** = $p < 0.001$, **** = $p < 0.0001$.

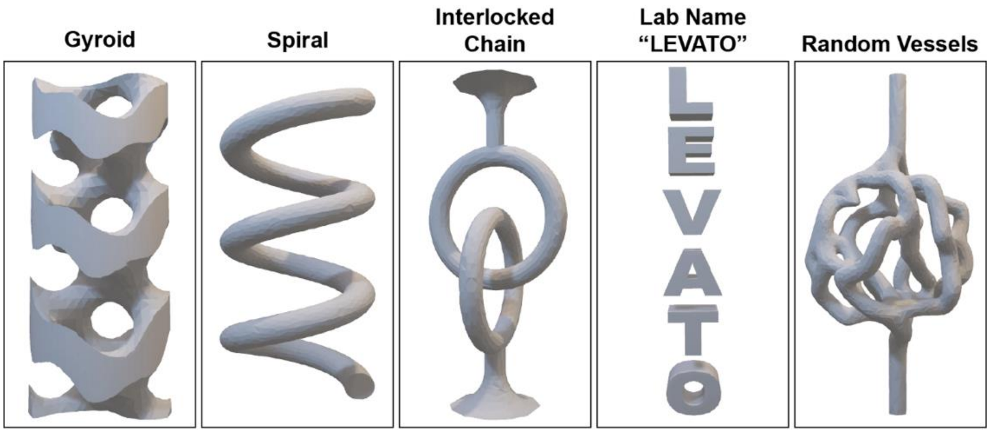

Supplementary Figure S9: STL models of the structures grafted onto gelNOR hydrogels in **Figure 5**. A gyroid, spiral, interlocked chain, lab name "LEVATO", and a random vessel-like network.

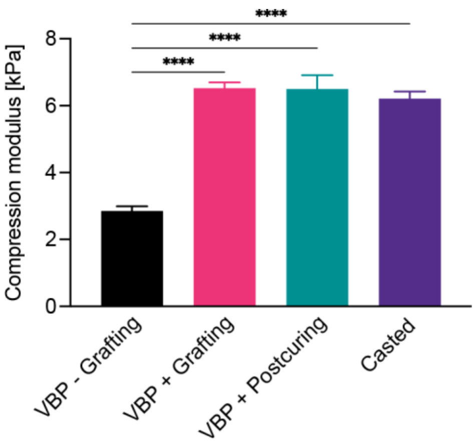

Supplementary Figure S10. Compression modulus of volumetrically printed 5 w/v% gelNOR hydrogels using DTT as crosslinker in a 1:1 thiol-ene ratio with 0.1 w/v% LAP as photoinitiator. Comparison of the compression modulus between non-grafted, grafted with Cy3-PEG-SH (750

mJ/cm^2), and post-cured (750 mJ/cm^2) and casted samples (n = 3) in absence of the thiolated PEG probe. **** = $p < 0.0001$.

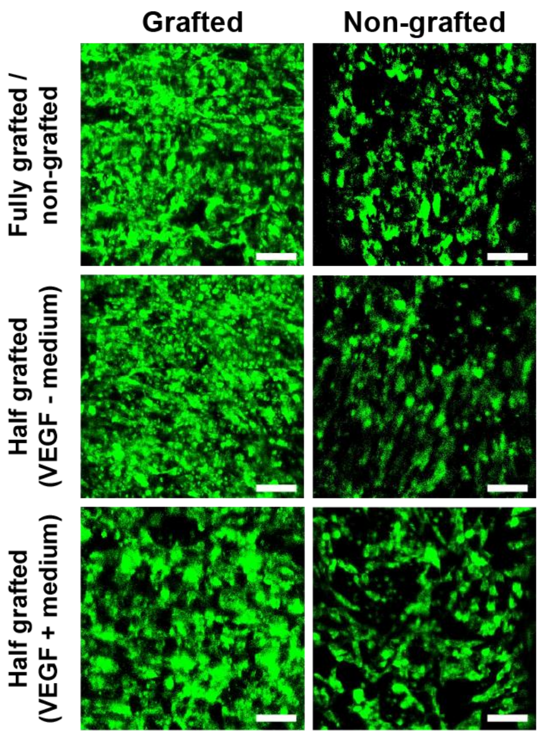

Supplementary Figure S11: Representative confocal z-projection images of HUVECs (GFP-positive) seeded on portions of the lumen of the printed vascular chip model (n = 3 biological replicates). Scale bars = 100 µm.

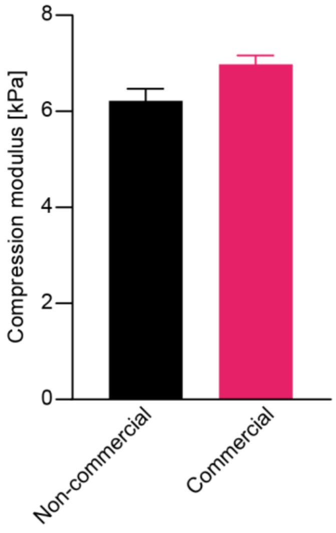

Supplementary Figure S12: Compression modulus comparison between commercially available gelNOR from BIO INX B.V. and custom synthesized non-commercially available gelNOR crosslinked under identical conditions (1:1 SH:NOR ratio using DTT as a crosslinker + 0.1 w/v% LAP). Young's modulus for both gels showed no significant differences (n = 5).

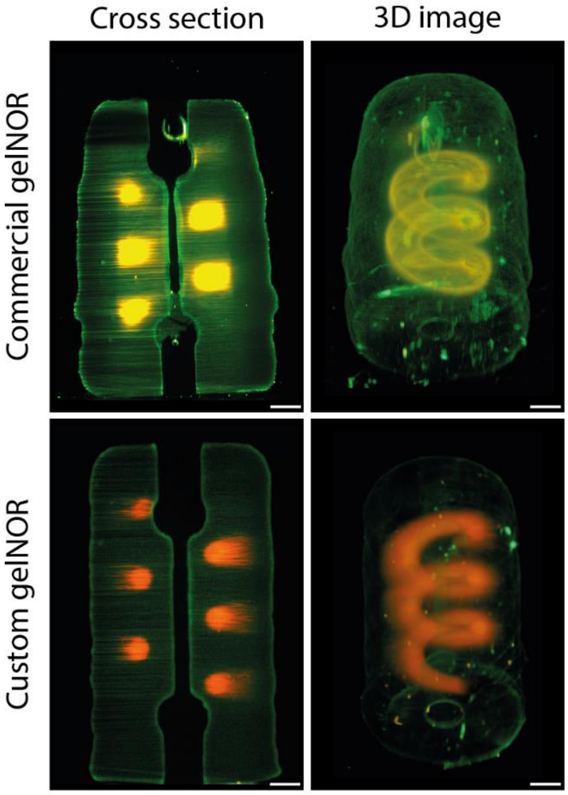

Supplementary Figure S13: Comparison of complex photografting of a spiral structure around a hollow channel, between commercially available gelNOR (supplied by BIO INX B.V.) and custom made gelNOR. Showing no differences in photografting application both in cross-sectional view as in 3D view. Samples were exposed to same infusing concentration (0.06 w/v% Cy3, 1.0 w/v% LAP, and 0.008 w/v% TEMPO) and same light dose (750 mJ/cm^2). Scalebar = 1 mm.

Supplementary Methods

Nuclear magnetic resonance (NMR): The ^{1}H-NMR spectrum of the gelatin-based materials was measured by NMR (400 MHz, Agilent 400 MR-NMR, Agilent technologies, USA). The material was dissolved in D_2O at a 4 w/v% concentration. NMR was not used to calculate the DoF of the material since a big overlap occurs with the material and the functionalized groups, leading to inaccurate calculation of the DoF following this method (n = 3 independently synthesized batches). NMR was used as a purity check of the material.

Cy3 concentration quantification: Cylindrical gelNOR (5% w/v, 1:1 thiol-ene ratio) samples with a volume of 100 µL were prepared. These samples were infused with the Cy3-PEG-SH infusion mix as described previously. The hydrogels were photografted at a dose of 750 mJ/cm^2. Samples were treated in 4 different conditions: 1) non-infused hydrogel, 2) infused hydrogel which was not grafted, 3) hydrogel which was infused and grafted and 4) freshly infused hydrogel without washing (to determine the initial loading concentration of Cy3-PEG-SH). After photografting samples were washed in PBS for 2 days at 4°C. Subsequently, the hydrogels were enzymatically degraded using 0.2 w/v% collagenase solution at 37°C for 1 hour. The fluorescent intensity for all conditions was measured with a CLARIOstar Plus® (BMG Labtech, Germany) plate reader with excitation at λ = 530 nm, and emission at λ = 580 nm. From this measurement, the concentration of fluorescent dye in the hydrogel formulation was calculated using a known standard curve (**Supplementary Figure S5**).
